# A predictive model of the cat cortical connectome based on cytoarchitecture and distance

**DOI:** 10.1007/s00429-014-0849-y

**Published:** 2014-07-26

**Authors:** Sarah F. Beul, Simon Grant, Claus C. Hilgetag

**Affiliations:** 1 Department of Computational Neuroscience, University Medical Center Hamburg-Eppendorf, 20246 Hamburg, Germany; 2 Division of Optometry and Visual Science, Henry Wellcome Laboratories for Visual Sciences, City University London, London, EC1V 0HB UK; 3 Department of Health Sciences, Sargent College, Boston University, Boston, MA 02215 USA

**Keywords:** Anatomical tract tracing, Cerebral cortex, Connectivity, Cytoarchitecture, Neuroinformatics

## Abstract

Information processing in the brain is strongly constrained by anatomical connectivity. However, the principles governing the organization of corticocortical connections remain elusive. Here, we tested three models of relationships between the organization of cortical structure and features of connections linking 49 areas of the cat cerebral cortex. Factors taken into account were relative cytoarchitectonic differentiation (‘structural model’), relative spatial position (‘distance model’), or relative hierarchical position (‘hierarchical model’) of the areas. Cytoarchitectonic differentiation and spatial distance (themselves uncorrelated) correlated strongly with the existence of inter-areal connections, whereas no correlation was found with relative hierarchical position. Moreover, a strong correlation was observed between patterns of laminar projection origin or termination and cytoarchitectonic differentiation. Additionally, cytoarchitectonic differentiation correlated with the absolute number of corticocortical connections formed by areas, and varied characteristically between different cortical subnetworks, including a ‘rich-club’ module of hub areas. Thus, connections between areas of the cat cerebral cortex can, to a large part, be explained by the two independent factors of relative cytoarchitectonic differentiation and spatial distance of brain regions. As both the structural and distance model were originally formulated in the macaque monkey, their applicability in another mammalian species suggests a general principle of global cortical organization.

**Electronic supplementary material:**

The online version of this article (doi:10.1007/s00429-014-0849-y) contains supplementary material, which is available to authorized users.

## Introduction

The intrinsic architecture of cortical and subcortical regions and their intrinsic and extrinsic connectivity patterns constitute the anatomical substrate for the elaborate information processing performed in the brain. Evidence accumulated from detailed quantitative studies of the connectome of cat, monkey and human cerebral cortex (Young 1992; Scannell et al. 1995, 1999; Hilgetag et al. 2000a; Kaiser and Hilgetag 2006; Zamora-López et al. 2010; Bassett et al. 2010; Modha and Singh 2010; Harriger et al. 2012; Goulas et al. 2014) has revealed a common topology that has been related to both behavioral measures and disease conditions in humans (Li et al. 2009; Fang et al. 2012). This topology, observed across several species, is characterized by dense connectivity among neighboring areas of the same major processing modules (visual, auditory, somato-motor, fronto-limbic), with relatively few direct long-range connections between them (Kaiser and Hilgetag 2006). Inter-modal integration is largely served by a collection of spatially delocalized hub-module areas, which possess widespread connections and are strongly interconnected among themselves, hence their designation as a ‘rich-club’ (Colizza et al. 2006; Zamora-López et al. 2011; Bullmore and Sporns 2012; Harriger et al. 2012). While the ‘rich-club’ is a costly feature in several aspects of cortical organization (Collin et al. 2013), including the disproportionate occupancy of white matter volume and associated high energy expenditure, this organization can also be considered functionally efficient for providing locally specialized (intra-modal) as well as longer-range (cross-modal) integration, and has been likened to the complex global infrastructure underlying human social and transport networks (Bassett and Bullmore 2006).

Nonetheless, the structural principles that govern the characteristic organization of global corticocortical connectivity remain elusive. There are several aspects of inter-areal cortical connections that need to be explained, such as their existence (i.e., the absence or presence of a connection), as well as their patterns of laminar origin and termination. Three main models have been proposed which consider different features of cortical organization as predictors for the characteristics of cortical connections.

First, cytoarchitectonic differentiation, measured principally in terms of the number and density of cellular layers, differs between the areas of the cerebral cortex (Brodmann 1909; von Economo and Koskinas 1925; Sanides 1970). Regularities in the interconnections of areas of distinct cytoarchitectonic differentiation have been observed (Rockland and Pandya 1979; Pandya and Yeterian 1985), and the *structural model* suggests that the laminar patterns of origins and terminations of inter-areal projections vary according to the relative cytoarchitectonic differentiation of the projection sources and targets (Barbas 1986; Barbas and Rempel-Clower 1997). Moreover, cytoarchitectonic differentiation has also been related to the existence and strength of corticocortical connections, such as within the visual module of cat cortex (Hilgetag and Grant 2010). To test the structural model, cortical cytoarchitecture is often operationalized by ranking cortical areas into architectural types, an ordinal measure which projects complex cortical structure into a single parameter (e.g., Barbas 1986; Barbas and Rempel-Clower 1997; Rempel-Clower and Barbas 2000; Barbas et al. 2005; Hilgetag and Grant 2010).

Second, the *distance model* proposes that the relative spatial position of areas across the cortex systematically influences the existence (Young 1992; Klyachko and Stevens 2003) and strength (Douglas and Martin 2007) of connections between them. Specifically, the model assumes that connections are more frequent, and more dense, among neighboring regions and sparser or absent between remote regions, an arrangement consistent with minimization of axonal wiring costs (Young 1992; Ercsey-Ravasz et al. 2013). Salin and Bullier (1995) further proposed that the laminar locations of projection origins and terminations also change gradually according to the physical distance between connected cortical regions.

Finally, in the *hierarchical model*, rankings of cortical areas have been constructed from the laminar origin and termination patterns of corticocortical projections (Felleman and Van Essen 1991; Scannell et al. 1995). These patterns were interpreted as directional information on projections, for example, ‘forward’, ‘backward’ and ‘lateral’ (Rockland and Pandya 1979; Felleman and Van Essen 1991), and hierarchical rankings were constructed as to fit projection directions with a minimal number of constraint violations (Hilgetag et al. 1996, 2000b; Reid et al. 2009). The level differences separating source and target areas in such hierarchies were then related to the areas’ connectivity, in particular quantitative measures of the relative distribution of projection origins in the upper and deep cortical layers (Barone et al. 2000; Vezoli et al. 2004).

Here, we test these three models on an extensive collation of corticocortical connectivity in the cat cerebral cortex compiled by Scannell et al. (1995). This data set comprises results of numerous anatomical tracing experiments, the traditional standard for measuring cortical connectivity, and has been utilized by several research groups to investigate structural and dynamic properties of the cat cortical connectome (Müller-Linow et al. 2008; Zamora-López et al. 2009, 2010; Gomez-Gardenes et al. 2010; Zamora-López et al. 2011; Tang et al. 2012; Bailey et al. 2013; de Reus and van den Heuvel 2013). One reason for this popularity is that the data set collates data from direct anatomical methods for tracing cortical connections in both anterograde and retrograde directions. The spatial resolution (at the level of individual cells and synapses) and reliability of this approach exceed that of indirect diffusion-based tractography methods (Alger 2012; Griffa et al. 2013).

Importantly, the conceptual models outlined above that we examine here have been developed and tested extensively for connections of the visual (Young 1992; Barone et al. 2000; Vezoli et al. 2004; Douglas and Martin 2007) and prefrontal cortex of the macaque monkey (Barbas 1986; Barbas and Pandya 1989; Barbas and Rempel-Clower 1997; Klyachko and Stevens 2003; Barbas et al. 2005; Medalla and Barbas 2006). Thus, their application to connections spanning the whole cortex in a different species provides an excellent test of the models’ generality.

## Materials and methods

We first introduce three anatomical variables that are hypothesized to constrain cortical connectivity in the context of the structural, distance, and hierarchical models of cortical organization. Then, we present the available data set of cat corticocortical connectivity, and describe measures and procedures used in the analyses.

### Distance

To characterize the spatial separation of areas across the cortical sheet, we computed their *border distance*, which is a pragmatic and widely used measure (Young 1992; Barbas et al. 2005) for estimating inter-areal distance in the absence of reliable three-dimensional area coordinates (also see “Discussion”). As part of their connectivity collation, Scannell et al. (1995) published a spatial adjacency matrix for their parcellation that indicates common area borders (their Fig. 6). In some cases, there was an apparent mismatch between the information in this adjacency matrix and the parcellation shown in the paper (Scannell et al. 1995, their Fig. 1). In most of these cases, we gave priority to information from the matrix, except where the map was unambiguous. Specifically, the following changes were made to the spatial adjacency matrix: we removed adjacencies of area 17 with areas CGp and RS; and we added adjacencies of area 18 with areas 20a and 20b; of area CGa with areas 17, 4 and 6 m; of area SVA with areas 18, 20b and RS; of area SIV with area Ig and of area 4 g with area 6 m (Online Resource 1 provides a list of abbreviations used for area names, see Scannell et al. (1995) for further details). From the spatial adjacency relations, we calculated the shortest distances between all pairs of areas, Δ_dist_; that is, we determined the minimum number of borders separating any two areas within the cortical parcellation adopted by Scannell and colleagues.

### Structural type ranking

**Fig. 1:**
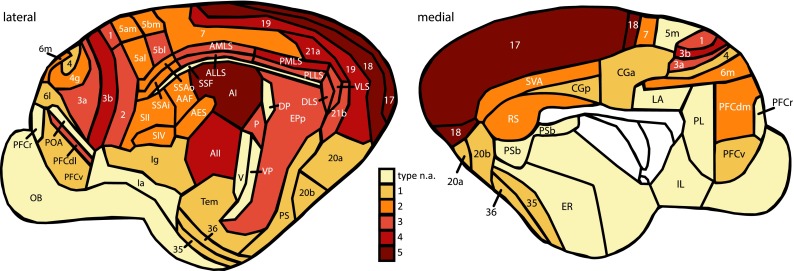
Parcellation of the cat cortex, adapted from Scannell et al. (1995). Areas were assigned to structural types 1–5 according to their level of cytoarchitectonic differentiation. *Type n.a.* no structural type was assigned. Abbreviations as in Online Resource 1

To evaluate the structural model, we computed the *structural type difference* between all pairs of areas. To this end, we rated cat cortical areas on an ordinal scale based on several criteria for their cytoarchitectonic differentiation, assigning a structural type to each area. One major feature was the relative width, density and granularization of layer IV (cf. Barbas 1986). Our classification thus follows the classical tradition of using cytoarchitectonic features for characterizing cortical areas as practiced since the early 20th century (Brodmann 1909; von Economo 1927). Modern techniques for quantification of cortical cytoarchitecture (e.g., by neuron density) have been applied to the macaque monkey (Dombrowski et al. 2001; Medalla and Barbas 2006), but such data were not available for the entirety of the cat cortex in the present study. We therefore determined structural types by qualitative criteria (also see “Discussion”). In our ranking procedure, first, areas of highest and lowest cytoarchitectonic differentiation were identified and assigned to the structural types 5 and 1, respectively. Second, areas in which cortical layers could be distinguished almost as well or as badly as in areas of types 5 and 1 were assigned the structural types 4 and 2, respectively. All remaining areas, necessarily of an intermediate differentiation, were assigned to structural type 3. For a more detailed description of the ranking procedure as well as photographic examples of structural types see Hilgetag and Grant (2010). Here we used these criteria to rank 49 areas across the whole cat cortex. Figure 1 depicts the assigned structural types in the cortical parcellation of Scannell et al. (1995). From our ranking scheme, we determined the difference between the structural types, Δ_type_ (cf. Barbas 1986), of any two of the 49 cortical areas for which we defined a structural type, where Δ_type_  =  type_source area_  −  type_target area_.

### Hierarchical level ranking

To evaluate the hierarchical model, we computed the *hierarchical level difference* between any two areas within the visual system. This analysis was confined to the visual module, because no equivalent hierarchical schemes exist for the other major modules of the cat cortical connectome. We used the hierarchy of the cat visual system as derived by Scannell et al. (1995, their Fig. 2) to determine the difference in hierarchical level, Δ_level_, where Δ_level_  =  level_source area_  −  level_target area_ (Barone et al. 2000; Hilgetag and Grant 2010). To exclude the possibility that our results hold only for this particular hierarchy, we alternatively computed Δ_level_ from the hierarchy of the cat visual system as proposed by Hilgetag et al. (2000b, their Fig. 12). In the analyses, we rectified an oversight in the published hierarchy diagram by reducing the level of area 17 to level 1, placing it on the same level as area 18.

### Projection data

Qualitative measures of corticocortical connections were extracted from an extensive collation of published reports of anatomical tract-tracing experiments in the cat (Scannell et al. 1995). The database that was provided for download in conjunction with the article includes 1,400 projections, which are mapped onto a parcellation consisting of 65 brain regions. The data set comprises the most complete summary of corticocortical connections in the cat to date. Even close to 20 years after its publication, this collation from 96 articles still represents the majority of anatomical tracing data available for this species, since few new tract-tracing results on the cat cortex have been published in the meantime. The data set has been widely interrogated (and cited 246 times to date, according to Web of Science, http://apps.webofknowledge.com).

#### Existence of projections

Existence of projections was given qualitatively as either absent (‘0’) or present, where the presence was described by ordinal weights as sparse (‘1’), intermediate (‘2’), or dense (‘3’). Importantly, projections weighted as ‘0’ were explicitly reported to be absent in the original literature, whereas no assumption was made about unknown projections (67 % of all potential projections among the areas). This distinction between absent and unknown projections was made in the companion database provided for download by Scannell et al. (1995), but not in the results published in the article itself. We conducted the majority of analyses on a version of the database converted to binary projection status, which rated projections as either absent or present and discarded information on projection density. This binarization enabled us to normalize projection frequencies across the tested variables, for example controlling for the fact that the data set contained information about a larger number of connections of distance 1 than of distance 5. An alternative approach for treating connection weights would have been to normalize projection frequencies separately for each ordinal density category. This approach would have yielded separate results for each density class, but not provided a comprehensive picture of the impact of the structural variables on connectivity overall.

For 954 of the 1,400 projections in the database (218 absent, 736 present) we were able to assess both Δ_dist_ and Δ_type_. For a subset of 308 projections (93 absent, 215 present), we could include additional information for Δ_level_ in the analyses. An overview of all available projection data together with the associated structural parameters is given in Online Resource 2.

Qualitative information on the presence or absence of connections is an undirected measure, as is the distance between two cortical areas, Δ_dist_. To meaningfully correlate these undirected variables with the directed variables Δ_type_ and Δ_level_, we reduced the latter two variables to their magnitude, that is, their absolute values, |Δ_type_| and |Δ_level_|.

To assess the distribution of present and absent projections across the considered anatomical variables, we calculated the cumulative percentage of present projections. For each anatomical variable, the cumulative percentage at each of its values was calculated as the sum of the number of present projections found up to this value, divided by the total number of present projections and multiplied by one hundred.

Relative projection frequencies were calculated separately for each value of each anatomical variable by considering all connections between pairs of areas at a particular distance, type difference or hierarchical level difference and assessing how many of them were indeed connected (e.g., determining the frequency of connections among all areas separated by 5 borders). Relative projection frequencies were, thus, calculated as the number of present projections with a specific value for a given anatomical variable, divided by the total number of examined projections (i.e. absent plus present projections) with that specific value.

#### Laminar projection patterns

Laminar projection patterns were available for a subset of 133 projections linking 22 cortical areas of the cat visual system. Scannell and colleagues classified the direction of projections as ‘ascending’, ‘lateral’, or ‘descending’ according to criteria laid out by Felleman and Van Essen (1991). Specifically, projections were classified as ‘ascending’, if they originated from the supragranular layers or in a bilaminar pattern from supra- and infragranular layers, and terminated predominantly in layer IV. ‘Lateral’ projections originated from both supra- and infragranular layers, and terminated in a columnar pattern throughout all cortical layers. ‘Descending’ projections originated either from infragranular layers or from both supra- and infragranular layers, and terminated in supra- and/or infragranular layers, avoiding layer IV in their terminations (Felleman and Van Essen 1991, their Fig. 3).

Based on this classification of projection directions, Scannell and colleagues derived an anatomical hierarchy of the cat visual system by arranging cortical areas such that a maximum number of ‘ascending’ projections pointed to higher levels and a maximum of ‘descending’ projections pointed to lower levels of the hierarchy.

The projection directions (Scannell et al. 1995; Hilgetag et al. 2000b, their Fig. 4) contain information on laminar projection origins and terminations in a pre-interpreted form. To assess the relationship between laminar projection patterns and structural factors, we used this set of 133 classified projections to calculate rank correlations of projection direction with Δ_type_ as well as Δ_level_. For these calculations, projection direction was consolidated in three categories: ‘ascending’, ‘lateral’, and ‘descending’. We included all projections whose direction classification had been marked as unreliable, due to insufficient or contradictory data (Hilgetag et al. 2000b, their Fig. 4), into the laminar categories that were indicated for them. For one projection analyzed by Scannell et al. (1995) and Hilgetag et al. (2000b), no Δ_type_ was available, because it targeted a region which had not been assigned a structural type. The present analyses were thus conducted on 132 projections.

The relation of projection direction to distance between cortical areas could not be evaluated using this data set, because distance is an undirected measure. Projection direction classified into three categories as used here, however, has no magnitude which could be evaluated independent of its direction, so that no meaningful combination of distance with an undirected adaptation of laminar projection patterns could be obtained.

### Topological measures

#### Modules of cortical areas

Zamora-López et al. (2010) used the database provided by Scannell et al. (1995) to analyze the connectivity of the entire cerebral cortex in the cat from a network-theoretical perspective and identified a ‘rich-club’ module of 11 hub areas, based on the internal density of links between high-degree nodes. The cortical areas constituting this hub meta-module were part of four other anatomical modules (visual, auditory, somatosensory-motor, and fronto-limbic) previously identified by different network-theoretical approaches (Scannell and Young 1993; Young 1993; Young et al. 1994; Hilgetag et al. 2000a; Sporns et al. 2004). These module classifications provide an opportunity to study the association between anatomical parameters and connection features at a larger-scale level of cortical organization. As Zamora-López and colleagues included only 53 of the 65 cortical areas of the original data set in their analyses, we restricted our analyses of the module features to the 48 areas which were both included in their analyses and possessed a structural type rating.

#### Node degree and weighted node degree

The node degree of a cortical area is the number of projections it takes part in. Here we added the number of afferent projections (in-degree) to the number of efferent projections (out-degree) for each area to obtain its overall node degree. Projections commonly comprise a strongly varying number of neurons, with projection strengths ranging over several orders of magnitude from only a few neurons to several thousand neurons (Scannell et al. 2000; Hilgetag and Grant 2000; Markov et al. 2011, 2014). We also computed node strength (the weighted node degree) by weighting each projection with its strength prior to summing up the present projections. As projection strength was rated ordinally in the data set provided by Scannell et al. (1995), we approximated the actual metric projection strength to vary over three orders of magnitude across sparse, intermediate and dense projections. We assigned weights of 10^0^, 10^1^, and 10^2^ to these respective descriptive categories to take into account the typical exponential distribution of projection densities (Hilgetag and Grant 2000; Markov et al. 2014). Moreover, we separately rank-correlated the number of projections with structural type for the projections of each ordinal strength.

#### Node connection range

We characterized the spatial range of the projections of cortical areas by assessing the distances of all afferent and efferent connections to and from each area, by computing the proportions of its projections formed by short (distance 1 and 2) as well as long (distance 4 and 5) connections, respectively. These proportions provided a simplified and robust measure of the projection distance profile of individual areas, from which we computed aggregate measures of node ranges for groups of areas.

### Statistical analyses

While type difference and border distance are inherently ordinal measures, projection directions can also be considered as ordinal values, by arranging them in the order of (‘ascending’, ‘lateral’, ‘descending’). To assess relations between these ordinal variables, we computed Spearman’s rank-correlation coefficient *ρ*. We also computed Spearman’s *ρ* to assess relations between relative projection frequencies and the respective anatomical variables.

To test two groups of ordinal measures for equality of their medians, we computed Wilcoxon rank sum test statistics (*W*). To test for equality of more than two groups of ordinal measures, we computed Kruskal–Wallis test statistics (*H*). We calculated Jonckheere–Terpstra test statistics (*JT*) to assess trends across multiple groups of ordinal measures. *JT* was computed using IBM SPSS Statistics Version 19 (IBM Corporation, Armonk, NY, USA). All tests were pre-assigned a two-tailed significance level *α* = 0.05. If not indicated otherwise, all analyses were performed using MATLAB Release 2012B (The MathWorks, Inc., Natick, MA, USA).

#### Linear discriminant analysis

To assess the distribution of present and absent projections across the variables |Δ_type_| and Δ_dist_ more closely, we performed a linear discriminant analysis (LDA) (Klecka 1980; Burns and Burns 2008). LDA determines a linear combination of predictive variables that optimally separates distinct classes of a dependent variable. Here, we used |Δ_type_| and Δ_dist_ as predictive variables, and existence of projections as the dependent variable. Given the non-significant correlation of relative projection frequencies with |Δ_level_| (see “Results”), we did not include |Δ_level_| into the LDA. We assumed uniform prior probabilities for the classes of the dependent variable (‘absent’ and ‘present’). LDA then provides a posterior probability for each combination of |Δ_type_| and Δ_dist_, which can be used to classify new data points (unknown connections) as either absent or present.

To account for the fact that not all combinations of the predictive variables can occur equally often (e.g., combinations of |Δ_type_| = 1 and Δ_dist_ = 1 are frequent in this cortical parcellation, while combinations of |Δ_type_| = 4 and Δ_dist_ = 4 are not), we normalized the numbers of absent and present projections of a specific combination of |Δ_type_| and Δ_dist_ by the maximally possible number of co-occurrences of that combination. This resulted in proportions %_absent_ and %_present_ of projections at each point in the predictive variable space. Note that %_absent_ + %_present_ ≠ 100, which reflects the fact that there is a remaining percentage of projections which have not been examined. To transform the resulting percentages into cases suitable as input for LDA, we constructed, for each combination of |Δ_type_| and Δ_dist_, *n*_a_ = %_absent_ cases with the respective values of the predictive variables and a dependent variable rating of ‘0’ (absent), and *n*_p_ = %_present_ cases with the same predictive variables but a dependent variable rating of ‘1’ (present). Compared to using the raw data as input for the LDA, this procedure adjusts the relative importance of examined projections by taking into account how thoroughly the underlying predictive variable space was sampled.

Cross-validation was performed by randomly excluding 10 % of the data from the training set and using this test set to validate the obtained model. We tested model performance at seven different classification thresholds, starting at 0.60 and increasing in 0.05 increments to 0.90. Connections were assigned the status ‘present’, if the posterior probability for the presence of connections at their associated |Δ_type_| and Δ_dist_ was equal to or larger than the classification threshold, and assigned the status ‘absent’, if their associated posterior probability was equal to or smaller than 1 minus the classification threshold (i.e., 0.40, decreasing in 0.05 increments to 0.10). We did not classify the status of connections with associated posterior probabilities that fell into the intermediate range. We performed 200 cross-validation cycles and report averaged results.

## Results

To test the structural, distance, and hierarchical models of cortical organization, we first assessed how informative they were regarding the presence or absence of interconnections between cortical areas, putting a special focus on the possibility of predicting connectivity. We then explored how cytoarchitectonic differentiation may relate to topological properties of cortical connectivity, such as membership in a ‘rich-club’ hub module or node degree. Finally, shifting perspective to further properties of the cortical connectome, we examined whether laminar projection patterns were well explained by structural type difference.

## Relationship of projection existence to anatomical variables

**Fig. 2:**
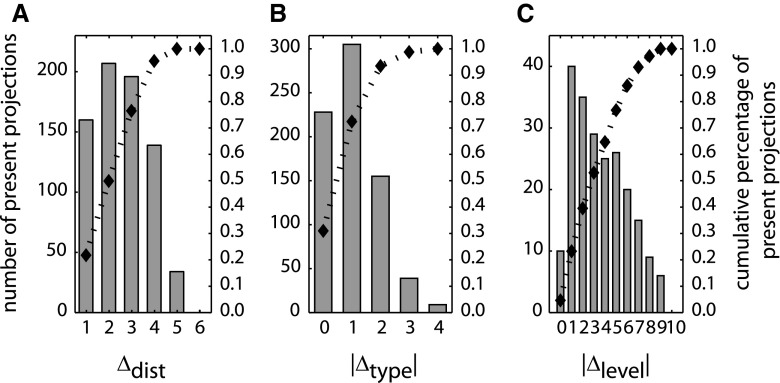
Cumulative percentages of present projections. For each anatomical variable, the absolute number of present projections is shown for each of its values (*bars*, *left axis*). Additionally, the cumulative percentage of present projections is indicated (*diamonds*, *right axis*). **a** Border distance Δ_dist_. **b** Absolute type difference |Δ_type_|. **c** Absolute hierarchical level difference |Δ_level_|

We evaluated the association among qualitative projection strength and the variables distance, Δ_dist_, structural type difference, Δ_type_, and hierarchical level difference, Δ_level_. Figure 2 shows the distribution of present projections for each parameter. It also depicts the cumulative percentage of present projections. About 75 % of present connections were found within values of Δ_dist_ = 1–3 (of the range 1–6 possible in the used cortical parcellation; Fig. 2a), within |Δ_type_| = 0–1 (of the range 0–4 possible between the 5 types; Fig. 2b), or within |Δ_level_| = 0–5 (of the range 0–10 occurring in this data set or 0–13 possible in the employed hierarchy; Fig. 2c). That is, the great majority of existing connections were short range and between areas of relatively similar intrinsic cytoarchitecture and hierarchical position.

**Fig. 3:**
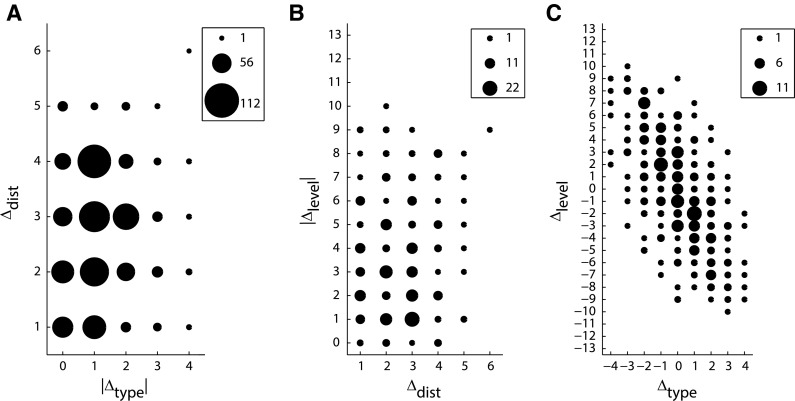
Interrelations of anatomical variables. **a** Distance Δ_dist_ was not correlated with absolute structural type difference |Δ_type_| or **b** with absolute hierarchical level difference |Δ_level_|. **c** Structural type difference Δ_type_ and hierarchical level difference Δ_level_ were strongly correlated. *Marker size* indicates number of projections

Rank-correlation analyses revealed no significant relationship between Δ_dist_ and |Δ_type_| (*ρ* = 0.06, *p* > 0.05, Fig. 3a), or between Δ_dist_ and |Δ_level_| (*ρ* = 0.04, *p* > 0.05, Fig. 3b), suggesting that Δ_dist_ was a largely independent factor. However, there was a strong correlation between Δ_type_ and Δ_level_ (*ρ* = −0.63, *p* < 0.001, Fig. 3c), discussed below.

**Fig. 4:**
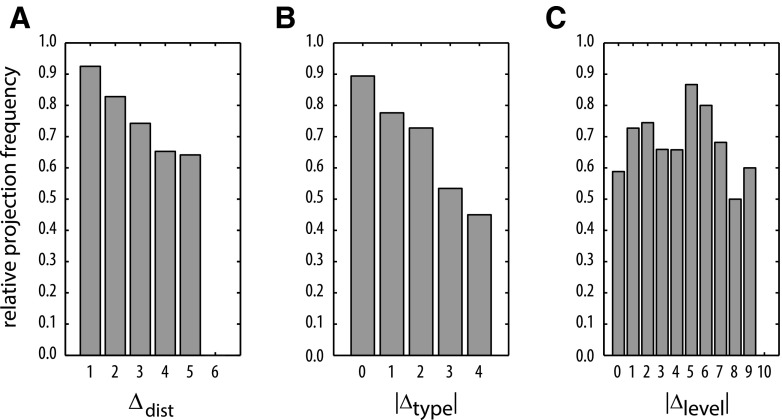
Correlation of anatomical variables with relative frequencies of present projections. **a**, **b** Distance Δ_dist_ and absolute structural type difference |Δ_type_| were negatively correlated with relative projection frequency. **c** Absolute hierarchical level difference |Δ_level_| was not correlated with relative projection frequency

Relative projection frequencies (i.e., relative proportions of present connections) were maximally negatively correlated with both Δ_dist_ (*ρ* = −1.00, *p* < 0.01, Fig. 4a) and |Δ_type_| (*ρ* = −1.00, *p* < 0.05, Fig. 4b). This monotonic decline for both factors indicates that the more distant or the more structurally dissimilar cortical areas are, the fewer projections are present between them. The results did not change substantially when the analyses were conducted only on the subset of 308 projections for which Δ_level_ was available (Δ_dist_: *ρ* = −1.0, *p* < 0.05, |Δ_type_|: *ρ* = −1.00, *p* < 0.05). By contrast, the relative proportion of present projections was not correlated with |Δ_level_| (*ρ* = −0.36, *p* > 0.05, Fig. 4c), indicating that the level difference between areas within the hierarchy proposed by Scannell et al. (1995) does not contain information about whether two areas are connected by an anatomical projection. Such a correlation was also absent for an alternative hierarchical ranking described by Hilgetag et al. (2000b) (see Methods: “Hierarchical level ranking”).

### Combination of structural type difference and distance

**Fig. 5:**
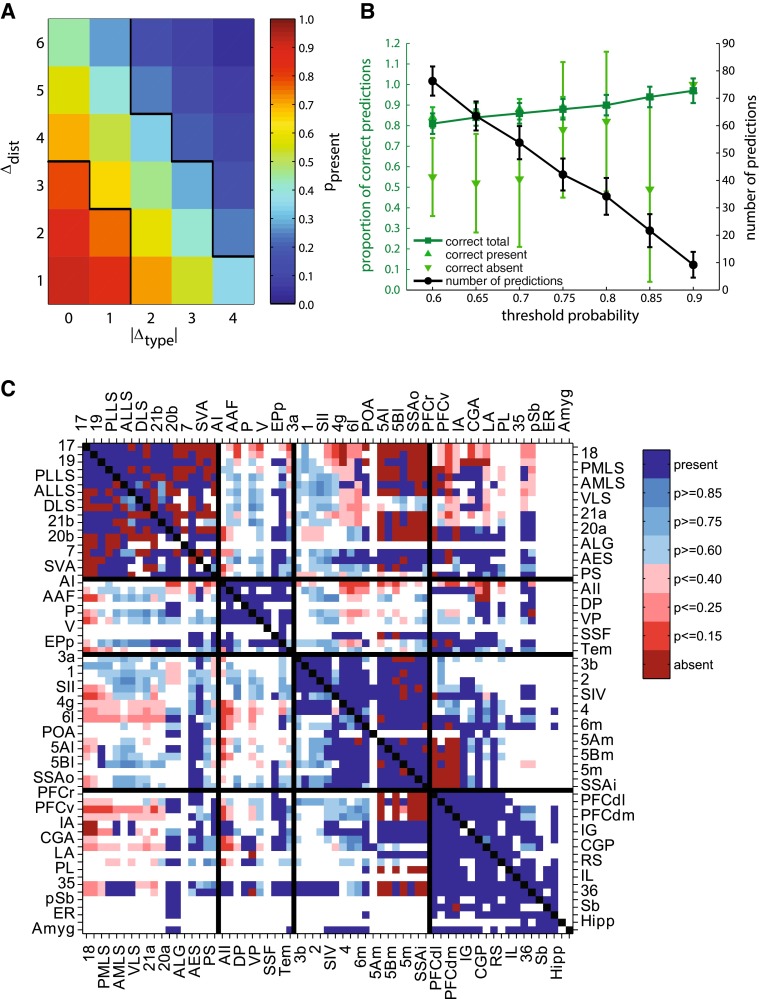
Results of linear discriminant analysis (LDA). **a** Posterior probabilities for presence of projections across the predictive variable space. *Black borders* enclose ranges of *p*_present_ > 0.75 and *p*_present_ <0.25. **b** Results of cross-validation at different prediction thresholds. Mean prediction accuracy for projections that were predicted to be present and absent (*light green*) as well as overall mean prediction accuracy (*dark green*) are shown. Mean number of predictions at each threshold is shown in *black*. *Error bars* indicate standard deviations. **c** Matrix of corticocortical connections in the cat, adapted from Scannell et al. (1995). Projections of known status are coded *dark red* (absent) and *dark blue* (present). Additionally, predicted connectivity for 926 unexamined projections is indicated. Projections predicted to be absent are shown in *lighter reds*, predictions predicted to be present are shown in *lighter blues*. *Color* saturation indicates how conservative a prediction threshold a particular prediction survived. *White cells* are unexamined connections for which no prediction has been made. The diagonal of intra-areal connections has been marked *black*. Projections’ source regions are arranged on the *vertical axis*, target regions are arranged on the *horizontal axis*. Abbreviations as in Online Resource 1. Note that area labels are split across *left*/*top* and *right*/*bottom axes*

We performed a LDA to distinguish between present and absent projections by their associated |Δ_type_| and Δ_dist_. The LDA assigned a significant contribution to classification performance to both variables, with standardized canonical discrimination function coefficients of 0.95 and 0.71 for |Δ_type_| and Δ_dist_, respectively. Figure 5a depicts the posterior probabilities that resulted from the LDA across the predictive variable space. Projections were confidently labeled as ‘present’ (*p*_present_ ≥ 0.75) if both |Δ_type_| and Δ_dist_ were in their lower range, that is |Δ_type_| < 2 and Δ_dist_ ≤ 3, and as ‘absent’ (*p*_present_ ≤ 0.25) if the variables were in their upper range of |Δ_type_| > 2 and Δ_dist_ ≥ 4.

From the posterior probabilities we made predictions about the existence of connections using different classification thresholds for the assignment of connections into the categories ‘present’ and ‘absent’. Figure 5b shows the cross-validated prediction accuracy of our model within the test sets across the used range of classification thresholds. Prediction accuracy increased as thresholds became more conservative, while at the same time the number of connections that were predicted decreased. A sensible choice for the classification threshold appeared to be *p*_present_ = 0.75 and *p*_present_ = 0.25 for ‘present’ and ‘absent’ connections, respectively. In this case the classification accuracy for both prediction categories exceeded 75 %, while the number of predictions remained substantial. These results illustrate how the combination of the two independent factors of absolute structural type difference and distance allowed us to confidently determine, for the subset of cortical connections that link cortical areas of appropriate |Δ_type_| and Δ_dist_, whether two cortical areas were connected. We therefore applied the posterior probabilities resulting from the model to predict the existence of connections that have not yet been investigated. Figure 5c depicts the classification of 926 as yet unexamined projections between cat cortical areas, where the classification threshold surpassed by the predicted connections is indicated by cell color saturation. At a classification threshold of 0.75 for present connections and 0.25 for absent connections, we made predictions about the existence of 418 unknown connections.

### Modules of cortical areas

**Fig. 6:**
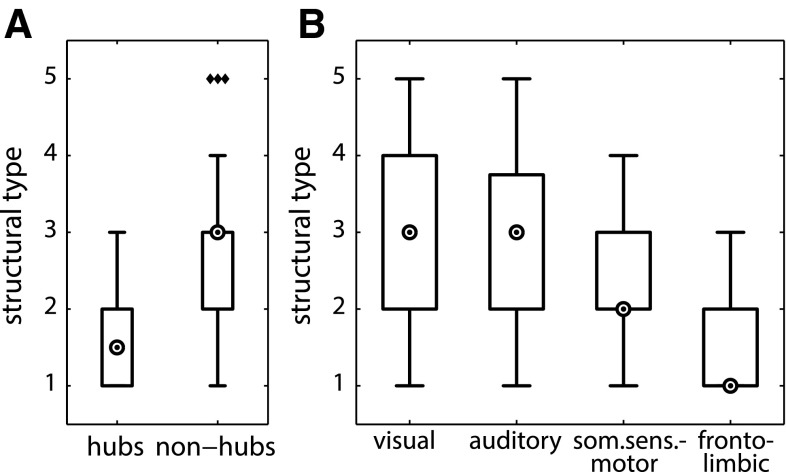
Distribution of structural types across modules of cortical areas. **a** Hub-module areas had a lower median type than non-hub-module areas. **b** Structural type gradually decreased across four anatomical modules of cortical areas. *Markers* inside *circles* indicate median degree, *diamonds* indicate outliers

The 11 cortical areas considered to constitute a ‘rich-club’ hub module by Zamora-López et al. (2010) had significantly lower structural types than all the remaining areas not belonging to the ‘rich-club’ (hub-module areas: median = 1.5, non-hub-module areas: median = 3; *W* = 146.5, *z* = −2.6, *p* = 0.01, Fig. 6a). Furthermore, the modality-specific clusters differed in their structural type medians (visual cluster: median = 3, auditory cluster: median = 3, somatosensory cluster: median = 2, fronto-limbic cluster: median = 1; *H*(3) = 11.1, *p* < 0.05, Fig. 6b). *Post hoc* tests, Bonferroni-corrected for multiple comparisons, revealed that the visual cluster had a higher median structural type than the fronto-limbic cluster (*W* = 255.0, *z* = 2.7, *p* = 0.0006, *α*_corr_ = 0.0008); all other pairwise differences in structural type between the four modality-specific clusters were not significant after correcting for multiple comparisons. However, structural type decreased gradually from the visual to the auditory, then to somatosensory and finally to the fronto-limbic cortices (*JT* = −2.0, *p* < 0.05).

### Node degree and weighted node degree

**Fig. 7:**
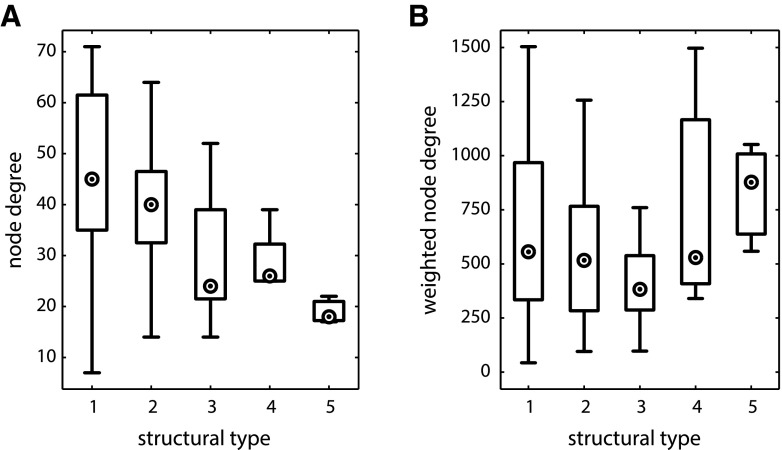
Degree distribution of cortical areas. **a** Node degree of cortical areas across structural types. **b** Weighted node degree of cortical areas across structural types. Node degree was correlated with structural type, while weighted node degree was not. *Markers* inside *circles* indicate median degree

The node degree (number of present projections) of cortical areas was negatively correlated with their structural type (*ρ* = −0.53, *p* < 0.001, Fig. 7a), such that areas with lower cytoarchitectonic differentiation had more connections. However, the weighted node degree (connection strength or density) of cortical areas was not correlated with their structural type (*ρ* = 0.004, *p* > 0.05, Fig. 7b). When calculated separately for projections of each ordinal projection strength, the correlation with structural type remained unaffected for sparse (*ρ* = −0.49, *p* < 0.001) and intermediate (*ρ* = −0.50, *p* < 0.001) projections, but disappeared for dense projections (*ρ* = 0.06, *p* > 0.05), thus explaining the lack of an overall correlation between structural type and node strength.

**Fig. 8:**
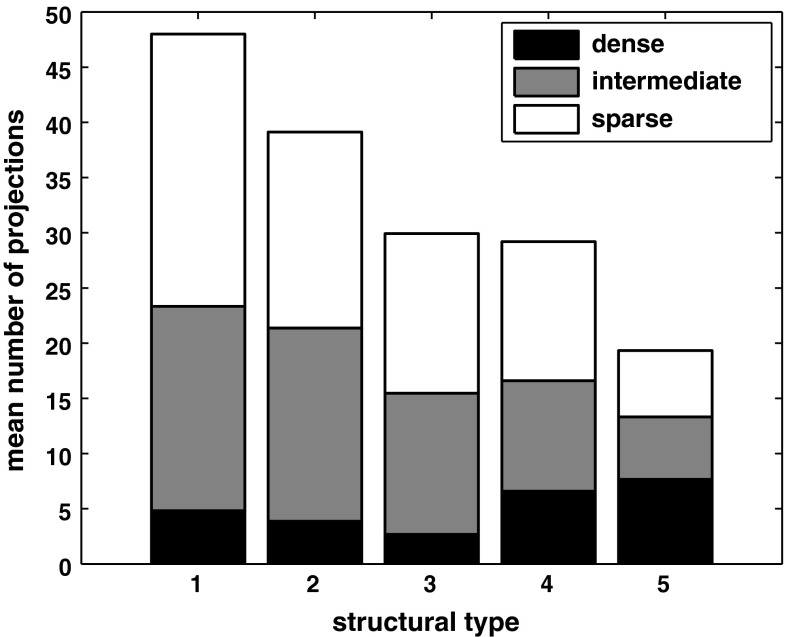
Mean number of projections across structural types. Means for ordinal projection strengths are indicated separately for each structural type. The maximal standard deviation across all structural types is 5 for the number of dense projections, 7 for the number of intermediate projections, and 9 for the number of sparse projections

We present this remarkable observation in a different form in Fig. 8, which depicts the mean number of dense, intermediate and sparse projections averaged across areas of a structural type. This representation underlines that the number of dense projections remains roughly constant, while the number of intermediate and sparse projections decreases notably with structural type, as revealed by the above correlation analyses.

Since the hub-module areas were originally identified, in part, by their very large number of connections and were found to be concentrated at the low end of the cytoarchitectonic differentiation spectrum, it is possible that the ‘rich-club’ module was mainly responsible for the strong association between high node degree and low structural type. To examine this possibility, we repeated the analyses with the ‘rich-club’ areas excluded. While this procedure had a quantitative impact, reducing the strength of the correlations, the relationship between low cytoarchitectonic differentiation and high node degree remained significant (*ρ* = −0.41, *p* < 0.01), and there was no qualitative effect on the lack of correlation with weighted node degree (*ρ* = 0.29, *p* > 0.05).

We observed an unexpected correlation between structural type and the total number of projections studied for a cortical area (comprising projections found to be absent as well as projections found to be present) (*ρ* = −0.40, *p* < 0.01). This effect raises the possibility that the correlation of node degree with structural type was a result of unequally distributed sampling efforts. However, it needs to be considered what impact additional data could have on the results. If all remaining unknown projections were to be examined, only a proportion of them would be found present. We verified that, if this proportion was equal across all structural types, the correlation we observed between node degree and structural type would remain moderate and significant up to an added proportion of present projections of 87 %. In the current data set, 77 % of examined projections were found to be present, whereas cortical connectivity levels have previously been estimated to reach about 50 % (Felleman and Van Essen 1991) or 66 % (Markov et al. 2014). Thus, even assuming the uncommonly high connectivity level of the examined data set (which likely reflects a lack of probing for absent projections in the literature, rather than a genuinely increased proportion of present projections), a uniform increase of present projections would still yield a correlation between node degree and structural type of *ρ* = −0.37, *p* < 0.01. A perhaps more probable proportion of present projections, such as 60 %, would result in a correlation of *ρ* = −0.44, *p* < 0.01. Thus, notwithstanding the possible undersampling of high structural type areas, our results suggest that areas of lower structural type are more frequently interconnected within the cortical connectome, and regardless of whether or not they are members of the ‘rich-club’ hub module.

### Node connection range

The projection distance profiles of cortical areas varied across structural types. When we compared aggregate node connection ranges for all areas of a given structural type across all five possible types, we found a positive relation for the proportion of short projections, such that areas of a higher structural type had higher proportions of short-range connections than areas of a lower structural type (*JT* = 3.1, *p* < 0.01). We also found an inverse relation between structural type and the proportion of long projections, such that areas of a lower structural type had a higher proportion of long projections than areas of a higher structural type (*JT* = −2.9, *p* < 0.01). For example, the average proportions of short- versus long-range connections for areas of the highest cytoarchitectonic differentiation (type 5) were 65 % and 9 %, respectively, compared to 45 % and 25 % for those of the lowest differentiation (type 1).

### Laminar projection profiles

**Fig. 9:**
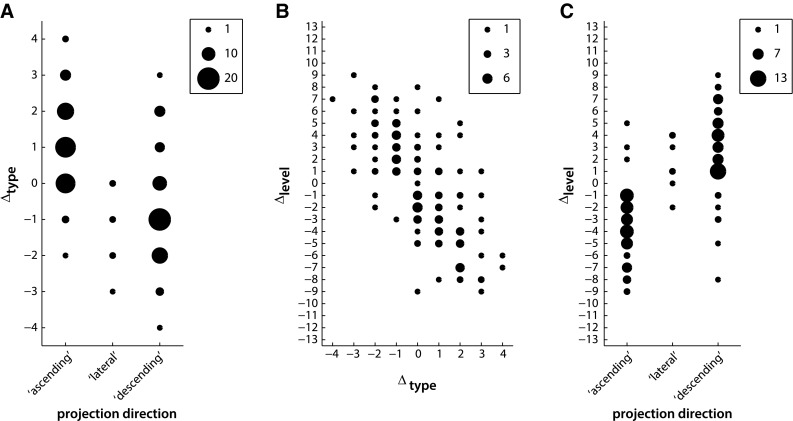
Correlation of anatomical variables with assigned directionalities of projections. **a** Structural type difference Δ_type_ was strongly correlated with projection directions and **b** hierarchical level difference Δ_level_. **c** Hierarchical level difference Δ_level_ was strongly correlated with projection directions. *Marker* size indicates number of projections

We investigated the relationship between the laminar projection patterns of connections, as coded in their assigned directions of ‘ascending’, ‘lateral’, and ‘descending’, and the associated Δ_type_, as well as Δ_level_. The Δ_type_ was strongly correlated with both projection direction (*ρ* = −0.53, *p* < 0.001, Fig. 9a) and Δ_level_ (*ρ* = −0.73, *p* < 0.001, Fig. 9b, compare also Fig. 3c). Projection direction was also strongly correlated with Δ_level_ (*ρ* = 0.74, *p* < 0.001, Fig. 9c), which was to be expected, as the hierarchical arrangements, and therefore the level differences, were derived from the projection directions in the first place. Results did not change if all projections classified as less reliable by Scannell et al. (1995) were excluded from the analysis.

## Discussion

**Fig. 10:**
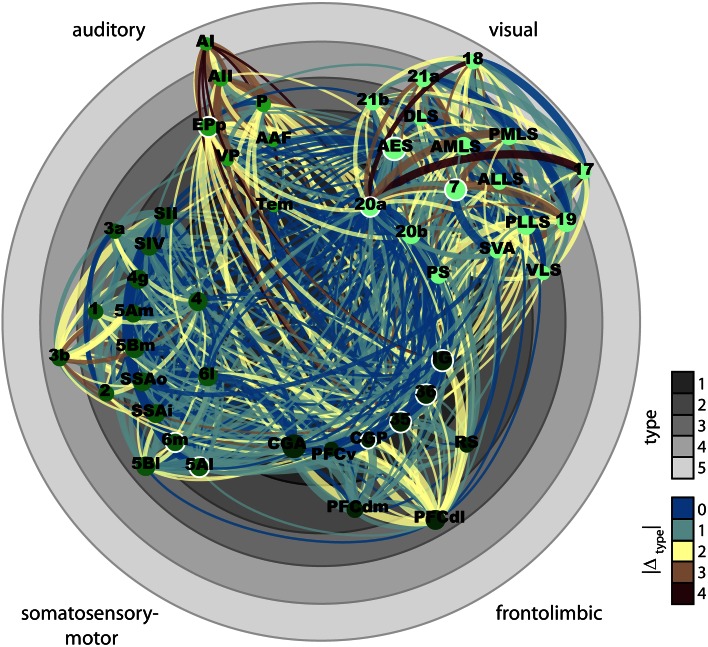
Visualization of the corticocortical connections collated in Scannell et al. (1995). All present projections between cortical areas for which a structural type was defined (49 of 65 areas) are displayed. *Circles* correspond to structural types, cortical areas are placed accordingly. Structural type increases from center to periphery. Projections are color-coded according to the absolute structural type difference of the connected areas. Ordinal projection strength (sparse, intermediate, or dense) is coded by increasing projection width. Nodes are grouped and *color-coded* according to anatomical modules as indicated. Node sizes indicate the areas’ (unweighted) degree. Hub-module areas, as classified by Zamora-López et al. (2010), are marked by a *white outline*

We used an extensive database (Scannell et al. 1995) of anatomical tracing experiments to assess, employing a variety of analytical approaches, the extent to which different anatomical variables associated with cortical organization can account for the local and global inter-areal connectivity of the cat cerebral cortex. Three anatomical factors were considered: differences between the cytoarchitectonic differentiation of cortical areas, particularly in the cellular density of cortical layers; border distances between areas; and their positions in the anatomical hierarchy originally constructed by Scannell and colleagues. There were four main findings: first, the relative cytoarchitectonic differentiation of areas, measured as structural type difference, contained significant information about several aspects of inter-areal corticocortical connectivity, including the existence (Fig. 4b) and laminar origin and termination patterns of projections (Fig. 9a). Second, the separation of areas across the cortical sheet, measured as border distance, also contained information about whether connections are present or not (Fig. 4a). Therefore, a linear combination of the two independent factors of structural type and distance allowed us to predict the existence of connections in the data set with more than 85 % accuracy at moderately conservative classification thresholds (Fig. 5b). Third, the relative position of areas in previously suggested hierarchical orderings, measured as level difference, was not informative about their inter-areal connectivity. Fourth, the cytoarchitectonic differentiation of areas was related to several of their topological properties. This included their membership in a densely connected ‘rich-club’ hub module as well as, more generally, the number of projections maintained by different areas (Fig. 7a) and the short- or long-range character of their connections (node connection range). Figure 10 summarizes these findings by depicting all existing connections in the data set between areas of determined structural type, with the brain regions arranged concentrically according to their structural type and clustered into the four major anatomical/functional modules of the cat cerebral cortex. Note that, already by visual inspection, connections of type difference 0 (blue) and 1 (blue-grey) clearly prevail, illustrating that most connections in the cat cerebral cortex link areas of similar cytoarchitectonic differentiation, and that areas of low cytoarchitectonic differentiation have the most connections.

### Methodological considerations

The present findings hinge on the reliability of the database and analyses employed. We used border distance to quantify the spatial separation of areas, rather than their Euclidean distance. This was partly because no detailed three-dimensional atlas quantifying the absolute distance between the mass centers of each area is currently available for the cat cortex. To obtain all the Euclidean area separations in the absence of such reliable information would thus have necessitated a number of unsubstantiated assumptions, whereas the use of border distances requires fewer constraints. Border distances are, however, potentially distorted by unequal area sizes and do not account for the actual projection lengths, as axons run under gyri and/or around sulci between their origins and destinations. Despite these complications, border distances generally correlate strongly with Euclidean distances where these latter are known (e.g., in macaque monkey visual cortex, our unpublished observation).

The connectivity database that we used comprises the most detailed information currently available about cat corticocortical connectivity, but some of its limitations warrant discussion. The database derives from anatomical studies published between 1968 and 1991 using intracellular transport of tracers. While this methodology usually enables the unambiguous detection of direct inter-areal connections, tracing studies are subject to technical caveats, which affect especially older results. For example, tracer uptake in fibers of passage can lead to false-positive results, while false-negative results can be caused by unsatisfactory tracer uptake, transport and/or detection (see Heimer and Robards 1981; Lanciego and Wouterlood 2011 for reviews). The database could therefore diverge from the actual pattern of connectivity especially by erroneous ‘absences’ of projections, which cannot be detected in a single tracing experiment. Notwithstanding these limitations, tract tracing remains the gold-standard technique for evaluating structural connections.

Another potential limitation is the adequacy of the specific cortical parcellation scheme used by the data collators, since alternative subdivisions have been proposed for all regions of cat cortex to that adopted by Scannell et al. (1995) which we followed here. The determination of area boundaries directly relates to connectivity patterns, with subtle differences in the latter often used to demarcate borders between neighboring areas. However, global organizational aspects of brain networks appear to be relatively robust to different parcellation schemes (de Reus and van den Heuvel 2013). The collators also necessarily averaged connectivity across areas, thus masking any inhomogeneities within and between them, such as possible differences in selective connectivity strengths between areas of the visual module containing ‘over-representations’ of central versus peripheral or of upper versus lower fields, or between tonotopic and non-tonotopic areas of auditory cortex. A further related question concerns the validity of the criteria used by the collators to assess relative inter-areal connection strengths (including apparent ‘absences’) across pathway tracing experiments that used techniques with differing sensitivities. While we thus acknowledge that future resolution of these matters may result in changes to our connectional summary (Fig. 10), we do not expect them to obscure the systematic properties of the global cortical connectome that we have identified. We would further note that, because the database contains information exclusively about ipsilateral corticocortical connections, our findings provide no insight into principles governing the connectivity across the cortical hemispheres.

### Relationships among anatomical variables

The absolute structural type difference and border distance of all pairs of areas were not correlated in our data set (Fig. 3a). This finding arose even though cytoarchitectonic differentiation frequently changes gradually across the cortical surface of cats (Hassler and Muhs-Clement 1964; Sanides and Hoffmann 1969) and primates (Sanides 1970; Barbas and Pandya 1989; Zilles and Amunts 2012), which intertwines structural type difference with the spatial distance between areas. However, the gradual change in cytoarchitectonic differentiation repeats multiple times across the cortical sheet, for instance, between primary and more remote ‘association’ areas within modules. In our approach, we assessed the border relationships of areas along all spatial directions, not just along a select axis (e.g., caudal to rostral), obscuring potential correlations for specific spatial gradients of cytoarchitecture. The resulting absence of a correlation thus indicates that the two factors of structural type and distance capture largely independent structural aspects at the global cortical level, justifying our treatment of them as independent variables. Moreover, border distance and hierarchical level difference were not correlated in our data set (Fig. 3b), indicating that they, too, describe independent aspects of cortical organization. In contrast, structural type difference and hierarchical level difference were found to be interdependent factors (Figs. 3c, 9b). This association arises inevitably from the fact that structural type differences and laminar patterns are strongly correlated and that cortical hierarchies are constructed from the laminar patterns, so that differences in hierarchical levels actually emerge from the strong relationship between cytoarchitectonic differentiation and laminar projection patterns.

### Structural model

Previous studies which were restricted largely to fronto-limbic regions of macaque monkey cortex (Barbas 1986; Barbas and Rempel-Clower 1997; Rempel-Clower and Barbas 2000; Barbas et al. 2005) or only to the visual module in the cat (Hilgetag and Grant 2010) have demonstrated strong associations between cytoarchitectonic differentiation and laminar connectivity. Our present findings, therefore, show that this anatomical principle extends across species and from the local, intra-modal level to the global organization of the cerebral cortex as a whole. Furthermore, the relative frequency by which two areas located anywhere in the cortex were linked by a direct anatomical projection decreased monotonically with their absolute difference in structural type (Fig. 4b), a result which concurs with previous findings for the cat visual cortex (Hilgetag and Grant 2010).

Assessing the hub-module areas identified by Zamora-López et al. (2010), we found that these areas were of a lower structural type than non-hub-module areas (Fig. 6a). Topological hubs, by definition, have a high node degree, that is, a large number of connections (Bullmore and Sporns 2009). We found, more generally, that there was a systematic inverse relationship between structural type and the number of connections across the whole data set, such that cortical areas of lower structural type had a higher node degree (Fig. 7a). More specifically, areas of a lower type appeared to possess a larger number of sparse and intermediate projections added to a backbone of dense connections which remains uniform across areas of all structural types (Fig. 8). We also found a relationship between structural type and the distances profile of areas, such that areas of a lower structural type had larger proportions of long connections and smaller proportions of short connections than observed in areas of a higher structural type. Thus, areas of a lower structural type appear to be more widely interlinked with other brain regions, both in terms of the number and the spatial range of their connections, compared to regions of higher structural type which typically correspond to the primary and immediately neighboring areas of each major uni-modal module.

Concerning the distribution of structural types within the four major uni-modal cortical communities (Scannell et al. 1995; Hilgetag et al. 2000a; Hilgetag and Kaiser 2004), we found a systematic variation in median structural type across the clusters, with the lowest median structural type in the fronto-limbic module and the highest in the visual module (Fig. 6b). This difference in average degree of cortical differentiation in different modules may partly explain their strong intra-modular connections—since minimal structural type differences are associated with dense connectivity between areas (Fig. 10)—and ultimately the separation of corticocortical connections into modular subnetworks linking areas of different sensory and motor functions. However, the actual mechanisms leading to the formation of cortical modules are still unresolved (Kaiser and Hilgetag 2007).

Generally, the principles governing the intriguing relationships among structural type, degree distribution and module location in the cerebral cortex are still unclear. A tentative explanation might be that these factors are developmentally interrelated. Higher node degree could, for example, be mediated by the relative time windows of the development of different areas, with lower-type areas appearing earlier in development than higher-type cortices and thereby being able to connect more widely and frequently with newly emerging areas. A similar mechanism has been proposed to account for the degree distribution of single neurons in *Caenorhabditis elegans* (Varier and Kaiser 2011; Towlson et al. 2013). Indeed, developmental evidence suggests that the time course of neurogenesis and cellular maturation in the mammalian cerebral cortex follows a broad rostral-to-caudal gradient (Sidman and Rakic 1973; Smart 1983; Smart et al. 2002), thus matching the lower-to-higher structural types and relative connectivity of frontal-to-occipital cortical regions.

One caveat applying to our structural type classification is that cytoarchitectonic differentiation of the mammalian cerebral cortex likely forms a gradual continuum (Sanides and Hoffmann 1969; Sanides 1970), as do laminar projection patterns within each of the three ordinal classes (Grant and Hilgetag 2005). Therefore, a measure objectively capturing gradual transitions across the cortex would be superior to the discrete structural types we assigned to brain regions. One such measure is neural density, which has been used by Medalla and Barbas (2006) to assess the structural model for projections of parietal to prefrontal cortices in the macaque monkey. Neuronal densities across cortical layers have previously been reported to vary systematically between areas classified into structural types by the criteria used here (Dombrowski et al. 2001). We are confident, therefore, that our discrete structural type classification captured genuine and relevant effects of cytoarchitectonic differentiation.

### Distance model

Due to the nature of the data, we could not evaluate the correspondence between (undirected) border distance and (directed) laminar projection patterns (see Methods: “Projection data”). However, we showed that pairs of areas are less frequently interconnected, the further they are separated across the cortical surface (Fig. 4a). This result is consistent with a large number of studies that investigated constraints of brain connectivity and found neural wiring length to be of critical importance (Bullmore and Sporns 2012). However, brain connectivity does not appear to be exclusively optimized with respect to physical wiring length, because trade-offs exist, for instance, with minimal topological path length (Kaiser and Hilgetag 2006; Bullmore and Sporns 2009). Thus, the distance model appears useful mainly as a predictor of the numerical neuron strength (high versus low) and existence (presence versus absence) of connections between neighboring versus widely separated cortical areas.

### Hierarchical model

Hierarchical level difference was strongly correlated with the assigned ‘hierarchical’ direction of projections (Fig. 9b). But this finding is neither surprising nor instructive, as the anatomical hierarchy had been constructed from these connection orientations in the first place (Scannell et al. 1995), so that the correlation between the two variables was based on a circular approach. Concerning the absence or presence of projections, the relative position of two areas within the hierarchical ordering was uninformative (Fig. 4c), with areas on adjacent levels of the hierarchy being no more frequently interconnected than those separated by more levels. This finding is contrary to the common understanding of hierarchical cortical schemes (Felleman and Van Essen 1991). It also resonates with several other shortcomings of hierarchical processing schemes, such as their failure to account for the level-skipping nature of many corticocortical (and thalamo-cortical) pathways (Symonds and Rosenquist 1984; Goldman-Rakic 1988; Mountcastle 1995; Hilgetag et al. 2000b; Petroni et al. 2001) or physiological features of cortical processing, in terms of near-synchronous response latencies (Nowak and Bullier 1997; Schmolesky et al. 1998) and similarities in receptive field size and complexity for the same stimulus (Hegdé and Van Essen 2007) at ‘lower’ and ‘higher’ hierarchical levels. Moreover, an optimal hierarchy has hitherto proven elusive, as large numbers of different orderings comply equally well with the constraints provided by the anatomical data (Hilgetag et al. 1996, 2000a). While the great laminar regularity of inter-areal projection patterns is certainly intriguing, it remains open for discussion whether elaborate schemes for ordering brain areas hierarchically are fundamentally helpful for understanding cortical organization (Hegdé and Felleman 2007; Markov et al. 2013).

### Predicting cortical projections

To integrate the information that the two independent parameters of structural type difference and border distance contain about the existence of projections, we combined them in a linear model. From this approach, we obtained a posterior probability of the existence of projections depending on both absolute structural type difference and distance (Fig. 5a). Projections between areas with both low absolute type difference and small border distance were very likely to be present, whereas the likelihood of a present projection strongly decreased once absolute type difference and border distance between areas grew larger. While there was considerable uncertainty about the status of projections between cortical areas possessing combinations of intermediate absolute type difference and intermediate border distance, we were able to derive predictions for the existence of as yet unstudied projections between cortical areas which fall into those ranges of absolute type difference and border distance which were confidently associated with either absence or presence of projections (Fig. 5c). Deliberate investigation of these currently unknown projections will allow gathering evidence to corroborate or contradict the structural and the distance model. Such data will also contribute to determining the relative importance of these two factors. Currently, the data set contains insufficient data to resolve the question of which of the factors dominates in cases of opposing predictions.

Our model predicts symmetric connectivity, that is, connections from areas of low to areas of high structural type are expected to be as likely as connections from high to low type. This prediction disregards the possibility that mechanisms may exist which preferentially mediate connections of one direction over the other, thus leading to asymmetric connectivity profiles. Furthermore, the data set provided an unequal sampling of the predictive variable space, which may have biased the resulting model. Nonetheless, our integrated model hints at a possible regularity, by revealing the high likelihood of corticocortical connections between areas of similar cytoarchitectonic differentiation, even across comparatively long distances. This finding is consistent with previous results indicating that neural networks are not optimized solely with respect to cost-conserving principles of reducing axonal wiring length (Kaiser and Hilgetag 2006), since connections across longer distances can provide network shortcuts that boost efficiency from a functional perspective. In the present study we did not explore the impact of potential functional constraints, such as topological path length (which may be related to functional efficiency), on connectivity features. Naturally, our approach for predicting the existence of connections could be augmented by considering additional functional or topological properties that have been explored previously (Jouve et al. 1998; Costa et al. 2007). Incorporating a broader range of factors could potentially enable us to reproduce features of the connectome that are not resolved by our model in its current form, such as modularity and hub features, which have been suggested to result from a combination of spatial and topological properties (Chen et al. 2013).

## Conclusion

Our study assessed models of corticocortical connectivity in the cat across a more comprehensive set of cortical areas and more functional modules than previous studies (Hilgetag and Grant 2010), considered topological area-based features, and integrated the relative cytoarchitectonic differentiation and spatial distance among areas into a predictive model of the global cortical connectome of the cat. We provide support for the structural model originally proposed by Barbas (1986), by showing that cytoarchitectonic differentiation contains information about different dimensions of brain connectivity, namely laminar patterns and the existence of inter-areal projections. Furthermore, the structural type of cortical areas appears to be related to their topological properties, for example, the degree of connectedness, with lower type (‘limbic’) areas possessing more connections. In addition, we found that the distance model also partly explains the existence of inter-areal connections. By contrast, our findings suggest that the hierarchical model has little explanatory value regarding the existence of inter-areal connections. In summary, relative cytoarchitectonic differentiation as well as spatial relations are good predictors of cortical connectivity in the cat brain, and can be combined to tentatively predict unexamined connections. While additional parameters remain to be tested for their impact on the cortical connectome, our results suggest that some of the general principles governing its organization have already been recognized.

## Electronic supplementary material

Below is the link to the electronic supplementary material.

Table of anatomical abbreviations (PDF 48 kb)

Table of projection data collation published in Scannell et al. (1995) and structural variables associated with each projection. See main text for further details. See Online Resource 1 for a list of abbreviations (PDF 137 kb)
