# Supplementary material for: A predictive model of the cat cortical connectome based on cytoarchitecture and distance
Source: Brain Struct Funct. 2014 Jul 26;220(6):3167–84. doi: 10.1007/s00429-014-0849-y (PMC4575693; doi:10.1007/s00429-014-0849-y)
Supplement: Supplementary file 1 — Table of anatomical abbreviations (PDF 48 kb) [file 429_2014_849_MOESM1_ESM.pdf]

# A Predictive Model of the Cat Cortical Connectome Based on Cytoarchitecture and Distance

Sarah F. Beul[1*], Simon Grant[2], Claus C. Hilgetag[1,3]

[1] Department of Computational Neuroscience, University Medical Center Eppendorf, 20246 Hamburg, Germany

[2] Division of Optometry and Visual Science, Henry Wellcome Laboratories for Visual Sciences, City University London, London EC1V 0HB, UK

[3] Department of Health Sciences, Sargent College, Boston University, Boston MA 02215, USA

* s.beul@uke.de

Brain Structure and Function

## Online Resource 1

Table of anatomical abbreviations.

| | |
|---|---|
| 1 | area 1 |
| 2 | area 2 |
| 4 | area 4 |
| 7 | area 7 |
| 17 | area 17 |
| 18 | area 18 |
| 19 | area 19 |
| 35 | area 35 |
| 36 | area 36 |
| 20a | area 20a |
| 20b | area 20b |
| 21a | area 21a |
| 21b | area 21b |
| 3a | area 3a |
| 3b | area 3b |
| 4g | area 4γ |
| 5al | lateral area 5a |
| 5am | medial area 5a |
| 5bl | lateral area 5b |
| 5bm | medial area 5b |
| 5m | medial area 5 |
| 6l | lateral area 6 |
| 6m | medial area 6 |
| AAF | anterior auditory field |
| AES | anterior ectosylvian sulcus |
| AI | primary auditory field |
| AII | secondary auditory field |
| ALG | anterolateral gyrus |
| ALLS | anterolateral lateral suprasylvian area |
| AMLS | anteromedial lateral suprasylvian area |

| | |
|---|---|
| Amyg | amygdala |
| CGa | anterior cingulate cortex |
| CGp | posterior cingulate cortex |
| DLS | dorsolateral suprasylvian area |
| DP | dorsoposterior auditory field |
| EPp | posterior part of the posterior ectosylvian gyrus |
| ER | entorhinal cortex |
| Hipp | hippocampus proper |
| Ia | agranular insula |
| Ig | granular insula |
| IL | infralimbic area |
| LA | anterior limbic cortex |
| P | posterior auditory field |
| PFCdl | dorsolateral prefrontal cortex |
| PFCdm | dorsomedial prefrontal cortex |
| PFCr | rostral prefrontal cortex |
| PFCv | ventral prefrontal cortex |
| PL | prelimbic area |
| PLLS | posterolateral lateral suprasylvian area |
| PMLS | posteromedial lateral suprasylvian area |
| POA | presylvian oculomotor area |
| PS | posterior suprasylvian area |
| pSb | presubiculum, parasubiculum, and postsubicular cortex |
| RS | retrosplenial cortex |
| Sb | subiculum |
| SII | second somatosensory area |
| SIV | fourth somatosensory area |
| SSAi | inner (deep) suprasylvian sulcal region of area 5 |
| SSAo | outer suprasylvian sulcal region of area 5 |
| SSF | suprasylvian fringe |
| SVA | splenial visual area |
| Tem | temporal auditory field |
| V | ventral auditory field |
| VLS | ventrolateral suprasylvian area |
| VP | ventroposterior auditory field |
