# Supplementary material for: A predictive model of the cat cortical connectome based on cytoarchitecture and distance
Source: Brain Struct Funct. 2014 Jul 26;220(6):3167–84. doi: 10.1007/s00429-014-0849-y (PMC4575693; doi:10.1007/s00429-014-0849-y)
Supplement: Supplementary file 2 — Table of projection data collation published in Scannell et al. (1995) and structural variables associated with each projection. See main text for further details. See Online Resource 1 for a list of abbreviations (PDF 137 kb) [file 429_2014_849_MOESM2_ESM.pdf]

A Predictive Model of the Cat Cortical Connectome Based on Cytoarchitecture and Distance

Sarah F. Beul[1*], Simon Grant[2], Claus C. Hilgetag[1,3]

[1] Department of Computational Neuroscience, University Medical Center Eppendorf, 20246 Hamburg, Germany

[2] Division of Optometry and Visual Science, Henry Wellcome Laboratories for Visual Sciences, City University London, London EC1V 0HB, UK

[3] Department of Health Sciences, Sargent College, Boston University, Boston MA 02215, USA

* s.beul@uke.de

Brain Structure and Function

Online Resource 2

Projection data collation published in Scannell et al. (1995) and structural variables associated with each projection. See main text for further details. See Online Resource 1 for a list of abbreviations.

Source area	Target area	Connection strength	Border distance	Type difference	Level difference
17	18	3	1	0	-1
17	PS	0	3	4	-3
17	VLS	2	4	2	-2
17	19	3	2	1	-3
17	PMLS	3	3	1	-4
17	SVA	0	1	3	-4
17	21a	2	3	1	-6
17	PLLS	1	4	2	-5
17	ALLS	0	4	2	-7
17	AMLS	2	3	2	-8
17	20a	3	2	4	-7
17	DLS	0	4	2	-8
17	21b	2	3	2	-8
17	20b	0	2	4	-9
17	7	0	2	3	-10
17	AES	0	5	3	-9
17	ALG	0	3	-	-
17	4g	0	3	3	-
17	6m	0	2	3	-
17	5Am	0	3	3	-
17	5Al	0	4	3	-
17	5Bm	0	3	3	-
17	5Bl	0	3	2	-
17	5m	0	2	-	-
17	SSAo	0	4	3	-
17	SSAi	0	4	3	-
18	17	3	1	0	1
18	PS	0	2	4	-2
18	VLS	1	3	2	-1
18	19	3	1	1	-2
18	PMLS	3	2	1	-3
18	SVA	0	1	3	-3
18	21a	2	2	1	-5
18	PLLS	1	3	2	-4
18	ALLS	1	3	2	-6
18	AMLS	3	2	2	-7
18	20a	2	1	4	-6
18	DLS	0	3	2	-7
18	21b	2	2	2	-7
18	20b	0	1	4	-8
18	7	1	1	3	-9
18	AES	0	5	3	-8
18	ALG	0	2	-	-
18	4g	0	3	3	-
18	5Am	0	3	3	-
18	5Al	0	3	3	-
18	5Bm	0	2	3	-
18	5Bl	1	2	2	-

18	5m	0	2	-	-
18	SSAo	0	3	3	-
18	SSAi	0	3	3	-
PS	17	0	3	-4	3
PS	18	0	2	-4	2
PS	19	1	2	-3	0
PS	PMLS	1	3	-3	-1
PS	SVA	1	2	-1	-1
PS	21a	1	2	-3	-3
PS	PLLS	3	3	-2	-2
PS	ALLS	0	3	-2	-4
PS	AMLS	1	4	-2	-5
PS	20a	2	1	0	-4
PS	21b	1	1	-2	-5
PS	20b	2	1	0	-6
PS	7	1	3	-1	-7
PS	CGA	1	3	0	-8
PS	AES	2	3	-1	-6
PS	35	1	2	0	-9
PS	36	2	1	0	-9
PS	SSF	2	2	-	-
PS	PFCr	0	4	-	-
PS	PFCdl	1	3	-2	-
PS	PFCv	1	3	0	-
PS	PFCdm	1	4	-1	-
PS	IG	2	2	0	-
PS	LA	1	4	-	-
VLS	17	0	4	-2	2
VLS	18	1	3	-2	1
VLS	19	1	2	-1	-1
VLS	PMLS	3	1	-1	-2
VLS	21a	0	1	-1	-4
VLS	PLLS	0	2	0	-3
VLS	20a	2	2	2	-5
VLS	DLS	2	1	0	-6
VLS	7	0	2	1	-8
VLS	CGA	1	3	2	-9
VLS	AES	1	4	1	-7
VLS	EPp	1	2	0	-
19	17	3	2	-1	3
19	18	3	1	-1	2
19	PS	0	2	3	0
19	VLS	2	2	1	1
19	PMLS	3	2	0	-1
19	SVA	3	2	2	-1
19	21a	3	1	0	-3

19	PLLS	2	3	1	-2
19	ALLS	1	3	1	-4
19	AMLS	2	2	1	-5
19	20a	3	1	3	-4
19	DLS	2	2	1	-5
19	21b	1	1	1	-5
19	20b	1	2	3	-6
19	7	2	1	2	-7
19	CGP	0	3	3	-7
19	CGA	0	2	3	-8
19	AES	0	5	2	-6
19	ALG	3	1	-	-
19	SSF	1	3	-	-
19	EPp	1	2	1	-
19	4g	0	4	2	-
19	6l	1	4	3	-
19	6m	0	3	2	-
19	5Am	0	3	2	-
19	5Al	1	3	2	-
19	5Bm	1	2	2	-
19	5Bl	2	2	1	-
19	5m	0	2	-	-
19	SSAo	0	3	2	-
19	SSAi	0	3	2	-
19	IA	0	4	-	-
19	IG	0	4	3	-
PMLS	17	3	3	-1	4
PMLS	18	2	2	-1	3
PMLS	PS	0	3	3	1
PMLS	VLS	3	1	1	2
PMLS	19	3	2	0	1
PMLS	SVA	0	3	2	0
PMLS	21a	1	1	0	-2
PMLS	PLLS	2	1	1	-1
PMLS	ALLS	0	2	1	-3
PMLS	AMLS	2	1	1	-4
PMLS	20a	3	3	3	-3
PMLS	DLS	0	2	1	-4
PMLS	21b	1	2	1	-4
PMLS	20b	0	3	3	-5
PMLS	7	0	1	2	-6
PMLS	CGA	1	2	3	-7
PMLS	AES	1	4	2	-5
PMLS	35	1	4	3	-8
PMLS	ALG	0	2	-	-
PMLS	6l	1	4	3	-

PMLS	5Am	0	3	2	-
PMLS	5Al	0	3	2	-
PMLS	5Bm	0	2	2	-
PMLS	5Bl	2	2	1	-
PMLS	5m	0	2	-	-
PMLS	SSAo	0	2	2	-
PMLS	SSAi	0	2	2	-
PMLS	PFCr	0	5	-	-
PMLS	PFCdl	0	5	1	-
PMLS	IA	1	4	-	-
PMLS	IG	1	4	3	-
SVA	17	0	1	-3	4
SVA	18	0	1	-3	3
SVA	19	3	2	-2	1
SVA	PMLS	1	3	-2	0
SVA	21a	0	3	-2	-2
SVA	PLLS	1	4	-1	-1
SVA	AMLS	1	3	-1	-4
SVA	20a	2	2	1	-3
SVA	DLS	0	4	-1	-4
SVA	20b	2	1	1	-5
SVA	7	3	2	0	-6
SVA	CGP	2	1	1	-6
SVA	CGA	2	1	1	-7
SVA	SSF	1	4	-	-
SVA	5Am	0	3	0	-
SVA	5Al	1	4	0	-
SVA	5Bm	0	3	0	-
SVA	5Bl	2	3	-1	-
SVA	5m	1	2	-	-
SVA	SSAo	2	4	0	-
SVA	LA	1	2	-	-
21a	17	3	3	-1	6
21a	18	3	2	-1	5
21a	PS	0	2	3	3
21a	VLS	0	1	1	4
21a	19	3	1	0	3
21a	PMLS	1	1	0	2
21a	SVA	0	3	2	2
21a	PLLS	1	2	1	1
21a	ALLS	0	3	1	-1
21a	AMLS	2	2	1	-2
21a	20a	2	2	3	-1
21a	DLS	0	2	1	-2
21a	21b	2	1	1	-2
21a	20b	0	3	3	-3

21a	7	2	1	2	-4
21a	AES	0	5	2	-3
21a	35	2	4	3	-6
21a	36	1	3	3	-6
21a	ALG	0	1	-	-
21a	EPp	1	2	1	-
21a	5Am	0	3	2	-
21a	5Al	1	3	2	-
21a	5Bm	0	2	2	-
21a	5Bl	2	2	1	-
21a	5m	0	2	-	-
21a	SSAo	0	3	2	-
21a	SSAi	0	3	2	-
PLLS	17	1	4	-2	5
PLLS	18	2	3	-2	4
PLLS	PS	3	3	2	2
PLLS	VLS	1	2	0	3
PLLS	19	1	3	-1	2
PLLS	PMLS	1	1	-1	1
PLLS	SVA	0	4	1	1
PLLS	21a	0	2	-1	-1
PLLS	ALLS	2	1	0	-2
PLLS	AMLS	1	2	0	-3
PLLS	20a	2	3	2	-2
PLLS	DLS	3	1	0	-3
PLLS	21b	0	2	0	-3
PLLS	20b	0	4	2	-4
PLLS	7	1	2	1	-5
PLLS	CGA	1	3	2	-6
PLLS	AES	3	3	1	-4
PLLS	35	1	5	2	-7
PLLS	ALG	0	3	-	-
PLLS	EPp	2	2	0	-
PLLS	6l	1	5	2	-
PLLS	6m	1	4	1	-
PLLS	5Am	0	4	1	-
PLLS	5Al	0	3	1	-
PLLS	5Bm	0	3	1	-
PLLS	5Bl	1	3	0	-
PLLS	5m	0	3	-	-
PLLS	SSAo	0	2	1	-
PLLS	SSAi	0	2	1	-
PLLS	PFCr	0	6	-	-
PLLS	PFCdl	1	5	0	-
PLLS	PFCv	0	5	2	-
PLLS	PFCdm	1	5	1	-

PLLS	IA	1	4	-	-
PLLS	IG	2	3	2	-
ALLS	17	0	4	-2	7
ALLS	18	0	3	-2	6
ALLS	PS	0	3	2	4
ALLS	VLS	1	3	0	5
ALLS	19	1	3	-1	4
ALLS	PMLS	0	2	-1	3
ALLS	21a	0	3	-1	1
ALLS	PLLS	2	1	0	2
ALLS	AMLS	1	1	0	-1
ALLS	20a	0	4	2	0
ALLS	DLS	2	2	0	-1
ALLS	20b	0	4	2	-2
ALLS	7	1	2	1	-3
ALLS	CGA	1	3	2	-4
ALLS	AES	2	3	1	-2
ALLS	SSF	1	1	-	-
ALLS	EPp	1	2	0	-
ALLS	6m	1	4	1	-
ALLS	5Am	0	3	1	-
ALLS	5Al	0	2	1	-
ALLS	5Bm	0	3	1	-
ALLS	5Bl	2	2	0	-
ALLS	5m	2	3	-	-
ALLS	SSAo	0	1	1	-
ALLS	SSAi	0	2	1	-
ALLS	PFCr	0	6	-	-
ALLS	PFCdl	1	5	0	-
ALLS	PFCv	0	5	2	-
ALLS	PFCdm	1	5	1	-
ALLS	IG	2	3	2	-
AMLS	17	3	3	-2	8
AMLS	18	3	2	-2	7
AMLS	PS	0	4	2	5
AMLS	19	2	2	-1	5
AMLS	PMLS	3	1	-1	4
AMLS	SVA	1	3	1	4
AMLS	21a	2	2	-1	2
AMLS	PLLS	0	2	0	3
AMLS	ALLS	1	1	0	1
AMLS	20a	0	3	2	1
AMLS	DLS	0	3	0	0
AMLS	20b	1	3	2	-1
AMLS	7	2	1	1	-2
AMLS	CGA	1	2	2	-3

AMLS	AES	1	3	1	-1
AMLS	ALG	1	2	-	-
AMLS	4g	1	4	1	-
AMLS	4	1	3	2	-
AMLS	6m	1	3	1	-
AMLS	5Am	1	3	1	-
AMLS	5Al	2	2	1	-
AMLS	5Bm	1	2	1	-
AMLS	5Bl	2	2	0	-
AMLS	5m	1	2	-	-
AMLS	SSAo	0	1	1	-
AMLS	SSAi	1	1	1	-
AMLS	PFCr	0	5	-	-
AMLS	PFCdl	0	5	0	-
AMLS	LA	1	3	-	-
20a	17	3	2	-4	7
20a	18	2	1	-4	6
20a	PS	0	1	0	4
20a	VLS	2	2	-2	5
20a	19	3	1	-3	4
20a	PMLS	3	3	-3	3
20a	SVA	0	2	-1	3
20a	21a	2	2	-3	1
20a	PLLS	1	3	-2	2
20a	ALLS	0	4	-2	0
20a	AMLS	0	3	-2	-1
20a	DLS	0	2	-2	-1
20a	21b	2	1	-2	-1
20a	20b	3	1	0	-2
20a	7	2	2	-1	-3
20a	CGP	2	3	0	-3
20a	AES	0	4	-1	-2
20a	35	2	2	0	-5
20a	36	1	2	0	-5
20a	ALG	0	2	-	-
20a	SSF	1	3	-	-
20a	EPp	2	2	-2	-
20a	6m	1	3	-1	-
20a	5Am	0	4	-1	-
20a	5Al	1	4	-1	-
20a	5Bm	0	3	-1	-
20a	5Bl	0	3	-2	-
20a	5m	0	3	-	-
20a	SSAo	0	4	-1	-
20a	SSAi	0	4	-1	-
20a	PFCr	0	5	-	-

20a	PFCdl	1	4	-2	-
20a	PFCv	1	4	0	-
20a	PFCdm	0	4	-1	-
20a	IA	1	3	-	-
20a	IG	1	3	0	-
20a	RS	2	2	-1	-
DLS	17	0	4	-2	8
DLS	18	0	3	-2	7
DLS	VLS	2	1	0	6
DLS	19	0	2	-1	5
DLS	PMLS	0	2	-1	4
DLS	SVA	0	4	1	4
DLS	21a	0	2	-1	2
DLS	PLLS	3	1	0	3
DLS	AMLS	0	3	0	0
DLS	20a	0	2	2	1
DLS	21b	1	1	0	0
DLS	20b	0	3	2	-1
DLS	7	0	3	1	-2
DLS	CGA	1	4	2	-3
DLS	AES	2	3	1	-1
DLS	EPp	2	1	0	-
DLS	IA	2	3	-	-
DLS	IG	2	3	2	-
21b	17	2	3	-2	8
21b	18	2	2	-2	7
21b	VLS	1	1	0	6
21b	19	1	1	-1	5
21b	PMLS	1	2	-1	4
21b	21a	2	1	-1	2
21b	PLLS	0	2	0	3
21b	AMLS	0	3	0	0
21b	20a	2	1	2	1
21b	DLS	1	1	0	0
21b	20b	0	2	2	-1
21b	7	2	2	1	-2
21b	35	2	3	2	-4
21b	36	1	2	2	-4
21b	EPp	1	1	0	-
21b	5Am	0	4	1	-
21b	5Al	1	4	1	-
21b	5Bm	0	3	1	-
21b	5Bl	2	3	0	-
21b	5m	0	3	-	-
21b	SSAo	0	4	1	-
21b	SSAi	0	3	1	-

20b	17	0	2	-4	9
20b	18	0	1	-4	8
20b	PS	2	1	0	6
20b	VLS	0	3	-2	7
20b	19	1	2	-3	6
20b	PMLS	0	3	-3	5
20b	SVA	2	1	-1	5
20b	21a	0	3	-3	3
20b	PLLS	0	4	-2	4
20b	ALLS	0	4	-2	2
20b	AMLS	1	3	-2	1
20b	20a	3	1	0	2
20b	DLS	0	3	-2	1
20b	7	2	2	-1	-1
20b	CGP	2	2	0	-1
20b	CGA	1	2	0	-2
20b	AES	1	4	-1	0
20b	35	2	1	0	-3
20b	36	1	1	0	-3
20b	SSF	1	3	-	-
20b	EPp	2	2	-2	-
20b	6m	1	3	-1	-
20b	5Am	0	4	-1	-
20b	5Al	1	4	-1	-
20b	5Bm	0	3	-1	-
20b	5Bl	0	3	-2	-
20b	5m	0	3	-	-
20b	SSAo	0	4	-1	-
20b	SSAi	0	4	-1	-
20b	PFCr	0	4	-	-
20b	PFCdl	1	3	-2	-
20b	PFCv	1	3	0	-
20b	PFCdm	0	4	-1	-
20b	IA	1	2	-	-
20b	IG	1	3	0	-
20b	LA	1	3	-	-
20b	RS	2	1	-1	-
7	17	0	2	-3	10
7	18	1	1	-3	9
7	PS	0	3	1	7
7	VLS	0	2	-1	8
7	19	2	1	-2	7
7	PMLS	0	1	-2	6
7	SVA	3	2	0	6
7	21a	2	1	-2	4
7	PLLS	1	2	-1	5

7	20a	1	2	1	3
7	DLS	0	3	-1	2
7	21b	1	2	-1	2
7	20b	0	2	1	1
7	CGP	3	2	1	0
7	CGA	1	1	1	-1
7	AES	0	4	0	1
7	35	1	3	1	-2
7	ALG	3	1	-	-
7	SSF	2	3	-	-
7	EPp	2	3	-1	-
7	4g	1	3	0	-
7	6l	2	3	1	-
7	6m	3	2	0	-
7	5Am	1	2	0	-
7	5Al	2	2	0	-
7	5Bm	2	1	0	-
7	5Bl	3	1	-1	-
7	5m	3	1	-	-
7	SSAo	2	2	0	-
7	SSAi	2	2	0	-
7	PFCr	1	4	-	-
7	PFCdl	1	4	-1	-
7	PFCv	0	4	1	-
7	PFCdm	1	3	0	-
7	IA	1	3	-	-
7	IG	1	4	1	-
7	LA	1	2	-	-
7	RS	2	3	0	-
CGP	20a	1	3	0	3
CGP	20b	1	2	0	1
CGP	7	3	2	-1	0
CGP	CGA	3	1	0	-1
CGP	AES	1	5	-1	1
CGP	DP	2	5	-	-
CGP	P	2	5	-2	-
CGP	SSF	1	5	-	-
CGP	EPp	3	4	-2	-
CGP	6m	3	2	-1	-
CGP	POA	2	4	-	-
CGP	PFCr	3	4	-	-
CGP	PFCdl	3	4	-2	-
CGP	PFCv	3	4	0	-
CGP	PFCdm	3	3	-1	-
CGP	IA	2	3	-	-
CGP	IG	2	4	0	-
CGP	LA	2	2	-	-
CGP	RS	1	1	-1	-
CGP	PL	1	3	-	-
CGP	pSb	1	2	-	-
CGA	20a	2	2	0	4
CGA	20b	2	2	0	2
CGA	AES	1	4	-1	2
CGA	ER	2	3	-	-
CGA	35	2	3	0	-1
CGA	36	1	3	0	-1
CGA	DP	2	5	-	-
CGA	P	2	5	-2	-
CGA	SSF	1	4	-	-
CGA	EPp	1	4	-2	-
CGA	6m	2	1	-1	-
CGA	5Am	1	2	-1	-
CGA	5Al	1	3	-1	-
CGA	5Bm	2	2	-1	-
CGA	5Bl	1	2	-2	-
CGA	5m	1	1	-	-
CGA	SSAo	2	3	-1	-
CGA	SSAi	1	3	-1	-
CGA	PFCr	3	3	-	-
CGA	PFCdl	3	3	-2	-
CGA	PFCv	2	3	0	-
CGA	PFCdm	3	2	-1	-
CGA	IA	2	2	-	-
CGA	IG	2	3	0	-
CGA	LA	2	1	-	-
CGA	RS	2	2	-1	-
CGA	PL	1	2	-	-
CGA	pSb	1	3	-	-
AES	17	0	5	-3	9
AES	18	0	5	-3	8
AES	PS	2	3	1	6
AES	19	0	5	-2	6
AES	PMLS	1	4	-2	5
AES	21a	1	5	-2	3
AES	PLLS	3	3	-1	4
AES	ALLS	2	3	-1	2
AES	AMLS	1	3	-1	1
AES	20a	2	4	1	2
AES	DLS	2	3	-1	1
AES	20b	2	4	1	0
AES	7	0	4	0	-1
AES	CGP	2	5	1	-1

AES	CGA	2	4	1	-2
AES	35	1	3	1	-3
AES	36	1	3	1	-3
AES	SSF	1	2	-	-
AES	EPp	1	3	-1	-
AES	SIV	2	1	0	-
AES	4g	2	5	0	-
AES	4	1	5	1	-
AES	6l	1	4	1	-
AES	6m	2	5	0	-
AES	POA	2	3	-	-
AES	5Am	2	4	0	-
AES	5Al	2	3	0	-
AES	5Bm	2	5	0	-
AES	5Bl	0	4	-1	-
AES	5m	2	4	-	-
AES	SSAo	2	3	0	-
AES	SSAi	1	2	0	-
AES	PFCr	1	4	-	-
AES	PFCdl	2	3	-1	-
AES	PFCv	0	3	1	-
AES	IA	1	2	-	-
AES	IG	2	1	1	-
ER	20a	2	2	-	-
ER	20b	2	1	-	-
ER	CGP	1	3	-	-
ER	CGA	1	3	-	-
ER	35	3	1	-	-
ER	36	2	2	-	-
ER	6m	1	3	-	-
ER	PFCr	1	4	-	-
ER	PFCv	2	3	-	-
ER	PFCdm	1	3	-	-
ER	LA	2	3	-	-
ER	RS	1	2	-	-
ER	PL	3	2	-	-
ER	IL	2	1	-	-
ER	pSb	2	1	-	-
ER	Sb	2	2	-	-
35	PMLS	1	4	-3	8
35	PLLS	1	5	-2	7
35	ALLS	1	5	-2	5
35	AMLS	1	4	-2	4
35	20a	1	2	0	5
35	20b	1	1	0	3
35	7	1	3	-1	2
35	CGP	1	3	0	2
35	CGA	2	3	0	1
35	ER	3	1	-	-
35	36	3	1	0	0
35	AI	1	4	-4	-
35	AII	2	3	-3	-
35	DP	2	4	-	-
35	P	2	4	-2	-
35	SSF	1	4	-	-
35	EPp	1	3	-2	-
35	Tem	1	2	0	-
35	3a	1	3	-2	-
35	3b	1	2	-3	-
35	1	1	3	-2	-
35	2	1	3	-2	-
35	SII	1	3	-1	-
35	SIV	2	3	-1	-
35	6m	2	4	-1	-
35	5Am	0	4	-1	-
35	5Al	0	4	-1	-
35	5Bm	1	4	-1	-
35	5Bl	0	4	-2	-
35	5m	1	3	-	-
35	SSAo	0	4	-1	-
35	SSAi	0	4	-1	-
35	PFCr	1	3	-	-
35	PFCdl	1	2	-2	-
35	PFCv	2	2	0	-
35	PFCdm	1	3	-1	-
35	IA	3	1	-	-
35	IG	1	2	0	-
35	LA	2	4	-	-
35	RS	1	2	-1	-
35	PL	2	3	-	-
35	IL	2	2	-	-
35	pSb	2	2	-	-
35	Sb	2	3	-	-
36	PS	2	1	0	9
36	PMLS	1	4	-3	8
36	SVA	1	2	-1	8
36	PLLS	1	4	-2	7
36	ALLS	1	4	-2	5
36	AMLS	1	4	-2	4
36	20a	1	2	0	5
36	20b	1	1	0	3
36	CGP	1	3	0	2

36	CGA	1	3	0	1
36	AES	2	3	-1	3
36	ER	2	2	-	-
36	35	3	1	0	0
36	AI	1	3	-4	-
36	AII	2	2	-3	-
36	DP	1	3	-	-
36	P	0	3	-2	-
36	SSF	1	3	-	-
36	EPp	2	2	-2	-
36	Tem	3	1	0	-
36	3a	1	3	-2	-
36	3b	1	2	-3	-
36	1	1	3	-2	-
36	2	1	3	-2	-
36	SII	1	3	-1	-
36	SIV	1	3	-1	-
36	6m	1	4	-1	-
36	5Am	0	4	-1	-
36	5Al	0	4	-1	-
36	5Bm	1	4	-1	-
36	5Bl	0	4	-2	-
36	5m	1	3	-	-
36	SSAo	0	4	-1	-
36	SSAi	0	4	-1	-
36	PFCr	1	3	-	-
36	PFCdl	1	2	-2	-
36	PFCv	2	2	0	-
36	PFCdm	1	3	-1	-
36	IA	1	1	-	-
36	IG	3	2	0	-
36	LA	2	4	-	-
36	RS	1	2	-1	-
36	PL	2	3	-	-
36	IL	2	3	-	-
36	pSb	2	2	-	-
ALG	17	0	3	-	-
ALG	18	0	2	-	-
ALG	19	3	1	-	-
ALG	PMLS	0	2	-	-
ALG	21a	0	1	-	-
ALG	PLLS	0	3	-	-
ALG	AMLS	1	2	-	-
ALG	7	3	1	-	-
AI	CGP	0	6	4	-
AI	35	1	4	4	-

AI	AII	2	1	1	-
AI	AAF	3	1	3	-
AI	DP	2	1	-	-
AI	P	2	1	2	-
AI	VP	2	2	3	-
AI	V	3	2	-	-
AI	SSF	1	1	-	-
AI	EPp	1	2	2	-
AI	Tem	1	2	4	-
AII	CGP	0	5	3	-
AII	AES	1	1	2	-
AII	35	2	3	3	-
AII	36	1	2	3	-
AII	AI	3	1	-1	-
AII	AAF	2	2	2	-
AII	P	2	1	1	-
AII	SSF	2	2	-	-
AII	EPp	2	2	1	-
AII	PFCr	1	4	-	-
AII	PFCdl	1	3	1	-
AII	PFCv	1	3	3	-
AII	PFCdm	1	4	2	-
AII	IA	2	2	-	-
AII	IG	2	1	3	-
AAF	CGP	0	5	1	-
AAF	CGA	0	4	1	-
AAF	AES	1	1	0	-
AAF	36	2	4	1	-
AAF	AI	3	1	-3	-
AAF	AII	2	2	-2	-
AAF	DP	2	2	-	-
AAF	P	2	2	-1	-
AAF	VP	1	3	0	-
AAF	V	2	3	-	-
AAF	SSF	2	1	-	-
AAF	EPp	1	2	-1	-
AAF	Tem	1	3	1	-
DP	20a	1	3	-	-
DP	20b	1	3	-	-
DP	CGP	2	5	-	-
DP	CGA	2	5	-	-
DP	35	2	4	-	-
DP	36	1	3	-	-
DP	AII	2	2	-	-
DP	P	2	1	-	-
DP	VP	1	2	-	-

DP	RS	2	4	-	-
P	20a	1	3	2	-
P	20b	1	3	2	-
P	CGP	2	5	2	-
P	CGA	2	5	2	-
P	35	2	4	2	-
P	36	0	3	2	-
P	AI	3	1	-2	-
P	AII	3	1	-1	-
P	AAF	1	2	1	-
P	DP	2	1	-	-
P	VP	2	1	1	-
P	V	2	1	-	-
P	EPp	2	1	0	-
P	Tem	1	2	2	-
P	RS	2	4	1	-
VP	CGP	1	5	1	-
VP	36	1	2	1	-
VP	AI	2	2	-3	-
VP	AII	2	2	-2	-
VP	AAF	1	3	0	-
VP	DP	2	2	-	-
VP	P	2	1	-1	-
VP	V	2	1	-	-
VP	EPp	2	1	-1	-
VP	Tem	2	1	1	-
V	EPp	1	2	-	-
SSF	PS	2	2	-	-
SSF	7	1	3	-	-
SSF	CGP	2	5	-	-
SSF	CGA	2	4	-	-
SSF	AES	2	2	-	-
SSF	AII	1	2	-	-
SSF	EPp	2	1	-	-
SSF	SIV	2	2	-	-
SSF	4g	1	5	-	-
SSF	4	1	5	-	-
SSF	6l	1	5	-	-
SSF	5Am	0	4	-	-
SSF	5Al	2	3	-	-
SSF	5Bm	0	4	-	-
SSF	5Bl	1	3	-	-
SSF	5m	1	4	-	-
SSF	SSAo	1	2	-	-
SSF	SSAi	2	1	-	-
SSF	PFCr	1	5	-	-

SSF	PFCdl	1	4	-	-
SSF	PFCv	1	4	-	-
SSF	PFCdm	1	5	-	-
SSF	LA	1	5	-	-
EPp	PS	2	1	2	-
EPp	21a	1	2	-1	-
EPp	20a	2	2	2	-
EPp	21b	1	1	0	-
EPp	20b	2	2	2	-
EPp	7	1	3	1	-
EPp	CGP	1	4	2	-
EPp	CGA	1	4	2	-
EPp	AES	1	3	1	-
EPp	35	2	3	2	-
EPp	36	1	2	2	-
EPp	AI	1	2	-2	-
EPp	AII	1	2	-1	-
EPp	P	2	1	0	-
EPp	VP	2	1	1	-
EPp	V	1	2	-	-
EPp	SSF	2	1	-	-
EPp	6m	2	5	1	-
EPp	PFCr	0	4	-	-
EPp	PFCdl	1	3	0	-
EPp	PFCv	0	3	2	-
EPp	PFCdm	1	4	1	-
EPp	IA	2	2	-	-
EPp	IG	2	2	2	-
EPp	LA	1	5	-	-
Tem	AII	2	1	-3	-
Tem	EPp	2	1	-2	-
Tem	PFCr	1	3	-	-
Tem	PFCdl	1	2	-2	-
Tem	PFCv	1	2	0	-
Tem	IA	2	1	-	-
Tem	IG	2	1	0	-
Tem	PL	2	3	-	-
3a	AES	1	4	1	-
3a	3b	2	1	-1	-
3a	1	2	2	0	-
3a	2	2	2	0	-
3a	SII	3	3	1	-
3a	4g	3	1	1	-
3a	4	2	1	2	-
3a	6l	2	1	2	-
3a	5Am	3	3	1	-

3a	5Al	3	3	1	-
3a	5Bm	0	3	1	-
3a	5Bl	0	3	0	-
3a	5m	0	2	-	-
3a	SSAo	1	3	1	-
3a	SSAi	2	3	1	-
3b	AES	1	3	2	-
3b	3a	2	1	1	-
3b	1	2	1	1	-
3b	2	2	1	1	-
3b	SII	3	2	2	-
3b	4g	2	2	2	-
3b	4	2	2	3	-
3b	6l	2	2	3	-
3b	5Am	2	2	2	-
3b	5Al	3	2	2	-
3b	5Bm	1	2	2	-
3b	5Bl	1	3	1	-
3b	5m	1	1	-	-
3b	SSAo	1	2	2	-
3b	SSAi	1	2	2	-
1	3a	2	2	0	-
1	3b	2	1	-1	-
1	2	2	1	0	-
1	SII	3	2	1	-
1	4g	2	3	1	-
1	4	1	3	2	-
1	6l	2	3	2	-
1	6m	1	3	1	-
1	5Am	3	1	1	-
1	5Al	1	2	1	-
1	5Bm	2	2	1	-
1	5Bl	0	3	0	-
1	5m	2	1	-	-
1	SSAo	1	2	1	-
1	SSAi	2	2	1	-
2	3a	2	2	0	-
2	3b	2	1	-1	-
2	1	2	1	0	-
2	SII	3	1	1	-
2	4g	2	3	1	-
2	4	1	3	2	-
2	6l	2	3	2	-
2	6m	1	3	1	-
2	5Am	2	2	1	-
2	5Al	2	1	1	-

2	5Bm	0	3	1	-
2	5Bl	2	2	0	-
2	5m	2	2	-	-
2	SSAo	2	1	1	-
2	SSAi	1	1	1	-
2	IA	1	2	-	-
2	IG	1	1	2	-
SII	AES	2	1	0	-
SII	35	1	3	1	-
SII	36	1	3	1	-
SII	3a	3	3	-1	-
SII	3b	3	2	-2	-
SII	1	3	2	-1	-
SII	2	3	1	-1	-
SII	SIV	1	1	0	-
SII	4g	3	4	0	-
SII	4	3	4	1	-
SII	6l	2	4	1	-
SII	6m	1	4	0	-
SII	POA	2	3	-	-
SII	5Am	2	3	0	-
SII	5Al	1	2	0	-
SII	5Bm	2	4	0	-
SII	5Bl	0	3	-1	-
SII	5m	2	3	-	-
SII	SSAo	0	2	0	-
SII	SSAi	3	1	0	-
SII	IA	1	2	-	-
SII	IG	1	1	1	-
SIV	CGA	1	4	1	-
SIV	AES	2	1	0	-
SIV	35	1	3	1	-
SIV	36	2	3	1	-
SIV	SSF	2	2	-	-
SIV	3b	1	3	-2	-
SIV	4g	1	5	0	-
SIV	4	1	5	1	-
SIV	6l	2	4	1	-
SIV	6m	2	5	0	-
SIV	POA	2	3	-	-
SIV	5Am	2	4	0	-
SIV	5Al	2	3	0	-
SIV	5Bm	2	5	0	-
SIV	5Bl	0	4	-1	-
SIV	5m	2	4	-	-
SIV	SSAo	1	3	0	-

SIV	SSAi	2	2	0	-
SIV	PFCr	0	4	-	-
SIV	PFCdl	1	3	-1	-
SIV	PFCv	1	3	1	-
SIV	PFCdm	1	4	0	-
SIV	IA	2	2	-	-
SIV	IG	2	1	1	-
SIV	LA	1	5	-	-
4g	CGA	2	2	1	-
4g	AES	1	5	0	-
4g	3a	2	1	-1	-
4g	3b	2	2	-2	-
4g	1	2	3	-1	-
4g	2	2	3	-1	-
4g	SII	2	4	0	-
4g	4	2	1	1	-
4g	6l	2	1	1	-
4g	6m	2	1	0	-
4g	5Am	3	4	0	-
4g	5Al	3	4	0	-
4g	5Bm	3	4	0	-
4g	5Bl	2	4	-1	-
4g	5m	2	3	-	-
4g	SSAo	2	4	0	-
4g	SSAi	2	4	0	-
4g	LA	2	2	-	-
4	CGA	2	1	0	-
4	AES	1	5	-1	-
4	ER	1	4	-	-
4	35	1	4	0	-
4	36	1	4	0	-
4	3a	2	1	-2	-
4	3b	2	2	-3	-
4	1	2	3	-2	-
4	2	2	3	-2	-
4	SII	2	4	-1	-
4	4g	2	1	-1	-
4	6l	2	2	0	-
4	5Am	2	3	-1	-
4	5Al	1	4	-1	-
4	5Bm	2	3	-1	-
4	5Bl	1	3	-2	-
4	5m	2	2	-	-
4	SSAo	1	4	-1	-
4	SSAi	1	4	-1	-
4	LA	2	2	-	-
6l	20a	1	4	0	-
6l	20b	1	4	0	-
6l	7	2	3	-1	-
6l	CGA	3	2	0	-
6l	EPp	1	4	-2	-
6l	SIV	2	4	-1	-
6l	6m	3	1	-1	-
6l	5Am	0	4	-1	-
6l	5Al	2	4	-1	-
6l	5Bm	1	4	-1	-
6l	5Bl	2	4	-2	-
6l	5m	1	3	-	-
6l	SSAo	1	4	-1	-
6l	SSAi	1	4	-1	-
6l	PFCr	1	1	-	-
6l	PFCdl	2	1	-2	-
6l	PFCv	1	2	0	-
6l	PFCdm	1	2	-1	-
6l	LA	2	2	-	-
6l	PL	2	2	-	-
6m	7	3	2	0	-
6m	CGP	3	2	1	-
6m	CGA	3	1	1	-
6m	AES	1	5	0	-
6m	ER	1	3	-	-
6m	35	1	4	1	-
6m	36	1	4	1	-
6m	EPp	1	5	-1	-
6m	SIV	2	5	0	-
6m	4g	1	1	0	-
6m	6l	3	1	1	-
6m	5Am	1	3	0	-
6m	5Al	1	4	0	-
6m	5Bm	1	3	0	-
6m	5Bl	2	3	-1	-
6m	5m	1	2	-	-
6m	SSAo	2	4	0	-
6m	SSAi	0	4	0	-
6m	PFCr	1	2	-	-
6m	PFCdl	2	2	-1	-
6m	PFCv	0	2	1	-
6m	PFCdm	2	1	0	-
6m	IA	2	3	-	-
6m	IG	2	4	1	-
6m	LA	2	1	-	-
6m	PL	2	1	-	-

6m	IL	2	2	-	-
POA	20a	1	4	-	-
POA	20b	1	3	-	-
POA	7	2	4	-	-
POA	CGP	2	4	-	-
POA	AES	2	3	-	-
POA	SII	1	3	-	-
POA	SIV	2	3	-	-
POA	6m	2	2	-	-
POA	IA	1	1	-	-
POA	IG	2	2	-	-
5Am	7	1	2	0	-
5Am	CGA	1	2	1	-
5Am	AES	1	4	0	-
5Am	SIV	2	4	0	-
5Am	4g	2	4	0	-
5Am	4	2	3	1	-
5Am	6l	0	4	1	-
5Am	6m	1	3	0	-
5Am	5Al	1	1	0	-
5Am	5Bm	3	1	0	-
5Am	5Bl	0	2	-1	-
5Am	5m	2	1	-	-
5Am	SSAo	2	2	0	-
5Am	SSAi	2	3	0	-
5Am	PFCr	0	5	-	-
5Am	PFCv	0	4	1	-
5Am	PFCdm	0	4	0	-
5Am	IA	1	3	-	-
5Am	LA	1	3	-	-
5Al	19	1	3	-2	-
5Al	20a	1	4	1	-
5Al	20b	1	4	1	-
5Al	7	2	2	0	-
5Al	CGA	1	3	1	-
5Al	AES	1	3	0	-
5Al	SII	1	2	0	-
5Al	SIV	2	3	0	-
5Al	4g	2	4	0	-
5Al	4	1	4	1	-
5Al	6l	2	4	1	-
5Al	6m	1	4	0	-
5Al	5Am	2	1	0	-
5Al	5Bm	1	2	0	-
5Al	5Bl	2	1	-1	-
5Al	5m	2	2	-	-
5Al	SSAo	2	1	0	-
5Al	SSAi	0	2	0	-
5Al	PFCr	0	5	-	-
5Al	PFCdl	1	4	-1	-
5Al	PFCv	0	4	1	-
5Al	PFCdm	0	5	0	-
5Al	IA	1	3	-	-
5Al	LA	1	4	-	-
5Bm	19	1	2	-2	-
5Bm	7	2	1	0	-
5Bm	CGA	1	2	1	-
5Bm	AES	1	5	0	-
5Bm	SII	2	4	0	-
5Bm	SIV	2	5	0	-
5Bm	4g	2	4	0	-
5Bm	4	2	3	1	-
5Bm	6l	1	4	1	-
5Bm	6m	1	3	0	-
5Bm	5Am	3	1	0	-
5Bm	5Al	2	2	0	-
5Bm	5Bl	2	1	-1	-
5Bm	5m	2	1	-	-
5Bm	SSAo	2	2	0	-
5Bm	SSAi	2	3	0	-
5Bm	PFCr	0	5	-	-
5Bm	PFCdl	0	4	-1	-
5Bm	PFCv	0	4	1	-
5Bm	PFCdm	0	4	0	-
5Bm	IA	1	3	-	-
5Bm	LA	1	3	-	-
5Bl	19	1	2	-1	-
5Bl	7	2	1	1	-
5Bl	CGA	1	2	2	-
5Bl	SSF	1	3	-	-
5Bl	4g	2	4	1	-
5Bl	4	1	3	2	-
5Bl	6l	2	4	2	-
5Bl	6m	2	3	1	-
5Bl	5Am	0	2	1	-
5Bl	5Al	3	1	1	-
5Bl	5Bm	2	1	1	-
5Bl	5m	2	2	-	-
5Bl	SSAo	2	1	1	-
5Bl	SSAi	1	2	1	-
5Bl	PFCr	0	5	-	-
5Bl	PFCdl	1	5	0	-

5Bl	PFCv	0	5	2	-
5Bl	PFCdm	0	4	1	-
5Bl	IA	1	4	-	-
5Bl	LA	1	3	-	-
5m	7	2	1	-	-
5m	CGA	1	1	-	-
5m	AES	1	4	-	-
5m	SSF	1	4	-	-
5m	SIV	1	4	-	-
5m	4g	2	3	-	-
5m	4	2	2	-	-
5m	6l	1	3	-	-
5m	6m	1	2	-	-
5m	5Am	2	1	-	-
5m	5Al	2	2	-	-
5m	5Bm	2	1	-	-
5m	5Bl	0	2	-	-
5m	SSAo	1	3	-	-
5m	SSAi	1	3	-	-
5m	PFCr	0	4	-	-
5m	PFCdl	0	3	-	-
5m	PFCv	0	3	-	-
5m	PFCdm	0	3	-	-
5m	IA	1	2	-	-
5m	LA	1	2	-	-
SSAo	7	2	2	0	-
SSAo	CGA	1	3	1	-
SSAo	AES	1	3	0	-
SSAo	SSF	1	2	-	-
SSAo	3b	1	2	-2	-
SSAo	SIV	1	3	0	-
SSAo	4g	2	4	0	-
SSAo	4	1	4	1	-
SSAo	6l	1	4	1	-
SSAo	6m	2	4	0	-
SSAo	5Am	3	2	0	-
SSAo	5Al	3	1	0	-
SSAo	5Bm	1	2	0	-
SSAo	5Bl	2	1	-1	-
SSAo	5m	2	3	-	-
SSAo	SSAi	2	1	0	-
SSAo	PFCr	0	5	-	-
SSAo	PFCdl	0	4	-1	-
SSAo	PFCv	0	4	1	-
SSAo	PFCdm	0	5	0	-
SSAo	IA	1	3	-	-
SSAo	LA	1	4	-	-
SSAi	7	1	2	0	-
SSAi	CGA	1	3	1	-
SSAi	AES	1	2	0	-
SSAi	SSF	2	1	-	-
SSAi	3b	1	2	-2	-
SSAi	SII	2	1	0	-
SSAi	SIV	2	2	0	-
SSAi	4g	2	4	0	-
SSAi	4	1	4	1	-
SSAi	6l	1	4	1	-
SSAi	5Am	2	3	0	-
SSAi	5Al	2	2	0	-
SSAi	5Bm	2	3	0	-
SSAi	5Bl	1	2	-1	-
SSAi	5m	2	3	-	-
SSAi	SSAo	1	1	0	-
SSAi	PFCr	0	5	-	-
SSAi	PFCdl	0	4	-1	-
SSAi	PFCv	0	4	1	-
SSAi	PFCdm	0	5	0	-
SSAi	IA	1	3	-	-
SSAi	LA	1	4	-	-
PFCr	7	1	4	-	-
PFCr	CGP	3	4	-	-
PFCr	CGA	3	3	-	-
PFCr	AES	1	4	-	-
PFCr	SSF	1	5	-	-
PFCr	5Am	0	5	-	-
PFCr	5Bm	0	5	-	-
PFCr	5m	0	4	-	-
PFCr	SSAo	0	5	-	-
PFCr	SSAi	0	5	-	-
PFCr	PFCdl	2	1	-	-
PFCr	PFCv	1	1	-	-
PFCr	PFCdm	2	1	-	-
PFCr	IA	2	2	-	-
PFCr	IG	2	3	-	-
PFCr	LA	1	3	-	-
PFCr	RS	2	5	-	-
PFCdl	PS	1	3	2	-
PFCdl	20a	1	4	2	-
PFCdl	20b	1	3	2	-
PFCdl	7	1	4	1	-
PFCdl	CGP	3	4	2	-
PFCdl	CGA	3	3	2	-

PFCdl	AES	1	3	1	-
PFCdl	SSF	1	4	-	-
PFCdl	EPp	1	3	0	-
PFCdl	5Am	0	4	1	-
PFCdl	5Al	1	4	1	-
PFCdl	5Bm	0	4	1	-
PFCdl	5Bl	1	5	0	-
PFCdl	5m	0	3	-	-
PFCdl	SSAo	0	4	1	-
PFCdl	SSAi	0	4	1	-
PFCdl	PFCr	3	1	-	-
PFCdl	PFCv	1	1	2	-
PFCdl	PFCdm	2	2	1	-
PFCdl	IA	2	1	-	-
PFCdl	IG	2	2	2	-
PFCdl	LA	2	3	-	-
PFCdl	RS	1	4	1	-
PFCdl	PL	2	2	-	-
PFCv	PS	1	3	0	-
PFCv	20a	1	4	0	-
PFCv	20b	1	3	0	-
PFCv	CGP	3	4	0	-
PFCv	CGA	2	3	0	-
PFCv	ER	1	3	-	-
PFCv	SSF	1	4	-	-
PFCv	EPp	1	3	-2	-
PFCv	5Am	0	4	-1	-
PFCv	5Bm	0	4	-1	-
PFCv	5m	0	3	-	-
PFCv	SSAo	0	4	-1	-
PFCv	SSAi	0	4	-1	-
PFCv	PFCr	2	1	-	-
PFCv	PFCdl	2	1	-2	-
PFCv	PFCdm	2	1	-1	-
PFCv	IA	2	1	-	-
PFCv	IG	1	2	0	-
PFCv	LA	1	2	-	-
PFCv	RS	1	4	-1	-
PFCv	IL	1	2	-	-
PFCdm	PS	1	4	1	-
PFCdm	7	1	3	0	-
PFCdm	CGP	3	3	1	-
PFCdm	CGA	3	2	1	-
PFCdm	ER	1	3	-	-
PFCdm	SSF	1	5	-	-
PFCdm	EPp	1	4	-1	-

PFCdm	5Am	0	4	0	-
PFCdm	5Bm	0	4	0	-
PFCdm	5m	0	3	-	-
PFCdm	SSAo	0	5	0	-
PFCdm	SSAi	0	5	0	-
PFCdm	PFCr	2	1	-	-
PFCdm	PFCdl	2	2	-1	-
PFCdm	PFCv	1	1	1	-
PFCdm	IA	2	2	-	-
PFCdm	IG	2	3	1	-
PFCdm	LA	2	2	-	-
PFCdm	RS	1	4	0	-
PFCdm	PL	2	1	-	-
PFCdm	IL	1	2	-	-
IA	17	0	3	-	-
IA	18	0	3	-	-
IA	PS	1	2	-	-
IA	PLLS	1	4	-	-
IA	ALLS	1	4	-	-
IA	CGP	2	3	-	-
IA	CGA	2	2	-	-
IA	AES	1	2	-	-
IA	ER	1	2	-	-
IA	35	3	1	-	-
IA	36	1	1	-	-
IA	AI	1	3	-	-
IA	AII	1	2	-	-
IA	EPp	2	2	-	-
IA	SII	1	2	-	-
IA	SIV	1	2	-	-
IA	6m	2	3	-	-
IA	5Am	1	3	-	-
IA	5Al	2	3	-	-
IA	5Bm	1	3	-	-
IA	5Bl	1	4	-	-
IA	5m	1	2	-	-
IA	SSAo	1	3	-	-
IA	SSAi	1	3	-	-
IA	PFCr	2	2	-	-
IA	PFCdl	2	1	-	-
IA	PFCv	2	1	-	-
IA	PFCdm	2	2	-	-
IA	LA	2	3	-	-
IA	PL	3	2	-	-
IA	IL	1	3	-	-
IG	17	0	4	-4	-

IG	18	0	4	-4	-
IG	PS	3	2	0	-
IG	PLLS	1	3	-2	-
IG	ALLS	1	3	-2	-
IG	7	1	4	-1	-
IG	CGP	2	4	0	-
IG	CGA	2	3	0	-
IG	AES	2	1	-1	-
IG	ER	1	3	-	-
IG	35	2	2	0	-
IG	36	3	2	0	-
IG	AI	1	2	-4	-
IG	AII	1	1	-3	-
IG	EPp	2	2	-2	-
IG	SII	1	1	-1	-
IG	SIV	2	1	-1	-
IG	4g	1	4	-1	-
IG	4	1	4	0	-
IG	6l	1	3	0	-
IG	6m	2	4	-1	-
IG	5Am	1	3	-1	-
IG	5Al	2	2	-1	-
IG	5Bm	1	4	-1	-
IG	5Bl	1	3	-2	-
IG	5m	1	3	-	-
IG	SSAo	1	2	-1	-
IG	SSAi	1	2	-1	-
IG	PFCr	2	3	-	-
IG	PFCdl	3	2	-2	-
IG	PFCv	1	2	0	-
IG	PFCdm	2	3	-1	-
IG	LA	2	4	-	-
IG	PL	2	3	-	-
IG	IL	2	4	-	-
LA	CGP	2	2	-	-
LA	CGA	2	1	-	-
LA	P	0	6	-	-
LA	5Am	1	3	-	-
LA	5Al	1	4	-	-
LA	5Bm	2	3	-	-
LA	5Bl	1	3	-	-
LA	5m	1	2	-	-
LA	SSAo	2	4	-	-
LA	SSAi	1	4	-	-
LA	PFCr	2	3	-	-
LA	PFCdl	2	3	-	-
LA	PFCv	1	2	-	-
LA	PFCdm	2	2	-	-
LA	IA	2	3	-	-
LA	IG	2	4	-	-
LA	RS	2	3	-	-
LA	PL	2	1	-	-
LA	IL	2	2	-	-
RS	20a	2	2	1	-
RS	20b	2	1	1	-
RS	CGP	1	1	1	-
RS	CGA	2	2	1	-
RS	ER	2	2	-	-
RS	35	2	2	1	-
RS	36	1	2	1	-
RS	DP	2	4	-	-
RS	P	2	4	-1	-
RS	PFCr	2	5	-	-
RS	PFCdl	1	4	-1	-
RS	PFCv	1	4	1	-
RS	PFCdm	1	4	0	-
RS	LA	2	3	-	-
RS	PL	1	4	-	-
PL	CGP	2	3	-	-
PL	CGA	1	2	-	-
PL	ER	1	2	-	-
PL	35	2	3	-	-
PL	36	1	3	-	-
PL	6m	2	1	-	-
PL	5Am	0	4	-	-
PL	5Bm	0	4	-	-
PL	5m	0	3	-	-
PL	SSAo	0	5	-	-
PL	SSAi	0	5	-	-
PL	PFCr	2	2	-	-
PL	PFCdl	1	2	-	-
PL	PFCv	1	1	-	-
PL	PFCdm	1	1	-	-
PL	IA	2	2	-	-
PL	LA	2	1	-	-
PL	IL	2	1	-	-
PL	Sb	1	4	-	-
IL	CGP	2	4	-	-
IL	CGA	2	3	-	-
IL	ER	2	1	-	-
IL	35	2	2	-	-
IL	36	1	3	-	-

IL	PFCr	1	3	-	-
IL	PFCdl	1	3	-	-
IL	PFCv	1	2	-	-
IL	PFCdm	1	2	-	-
IL	IA	1	3	-	-
IL	LA	2	2	-	-
IL	PL	2	1	-	-
IL	Sb	2	3	-	-
pSb	20a	1	2	-	-
pSb	20b	1	1	-	-
pSb	CGP	1	2	-	-
pSb	CGA	2	3	-	-
pSb	ER	3	1	-	-
pSb	35	2	2	-	-
pSb	36	2	2	-	-
pSb	PFCr	1	5	-	-
pSb	PFCv	1	4	-	-
pSb	PFCdm	1	4	-	-
pSb	LA	2	4	-	-
pSb	PL	2	3	-	-
pSb	IL	1	2	-	-
pSb	Sb	2	1	-	-
Sb	20a	1	3	-	-
Sb	20b	1	2	-	-
Sb	CGA	1	4	-	-
Sb	ER	2	2	-	-
Sb	PFCr	1	6	-	-
Sb	PFCdl	1	5	-	-
Sb	PFCv	1	5	-	-
Sb	PFCdm	0	5	-	-
Sb	IA	2	4	-	-
Sb	IG	1	5	-	-
Sb	LA	2	5	-	-
Sb	RS	2	2	-	-
Sb	PL	3	4	-	-
Sb	IL	3	3	-	-
Hipp	ER	3	3	-	-
Hipp	35	1	4	-	-
Hipp	pSb	2	2	-	-
Hipp	Sb	2	1	-	-
Amyg	20a	1	3	-	-
Amyg	20b	1	2	-	-
Amyg	CGA	1	4	-	-
Amyg	ER	2	1	-	-
Amyg	35	2	1	-	-
Amyg	36	2	2	-	-
Amyg	Tem	1	3	-	-
Amyg	SII	1	4	-	-
Amyg	SIV	1	4	-	-
Amyg	4g	1	4	-	-
Amyg	4	1	4	-	-
Amyg	6l	2	4	-	-
Amyg	6m	2	3	-	-
Amyg	PFCr	2	4	-	-
Amyg	PFCdl	2	3	-	-
Amyg	PFCv	2	3	-	-
Amyg	PFCdm	2	3	-	-
Amyg	IA	2	2	-	-
Amyg	IG	2	3	-	-
Amyg	LA	1	3	-	-
Amyg	PL	3	2	-	-
Amyg	IL	2	1	-	-
Amyg	pSb	2	2	-	-
Amyg	Sb	2	3	-	-
